# Arsenome, Arsenobolome, and Arsenobiolome

**DOI:** 10.3390/ijms262110761

**Published:** 2025-11-05

**Authors:** Fernando J. Pereira, Roberto López, A. Javier Aller

**Affiliations:** 1 Area of Analytical Chemistry, Department of Applied Chemistry and Physics, Faculty of Biological and Environmental Sciences, University of León, Campus de Vegazana, s/n, E-24007 León, Spain; 2 Area of Physical Chemistry, Department of Applied Chemistry and Physics, Faculty of Biological and Environmental Sciences, University of León, Campus de Vegazana, s/n, E-24007 León, Spain

**Keywords:** arsenic, carcinogenesis, DNA methylation

## Abstract

A complete characterisation of the potential biological implications of any chemical species requires assessing as much information as possible about the dose of all physicochemical forms involved in its metabolic pathways or any other biological activity (beneficial or harmful). Research investigating the biological significance of arsenic species in living systems needs to involve not only the chemical characterisation of the complete set of arsenic-containing species (arsenome), but also the distinction of all arsenic-bearing metabolites (arsenobolome) and those arsenic-containing species involved directly in specific beneficial or harmful processes (arsenobiolome). This work offers insight into the above considerations regarding arsenic species that are of toxicological significance. We highlight the differences in the metabolic and toxicological behaviour of inorganic (*i*As) and organoarsenic (*o*As) species, focusing on mechanistic clarification, particularly in signalling transduction, chronic effects, genotoxicity, and oxidative deoxyribonucleic acid (DNA) damage. The beneficial applications of arsenic compounds in the treatment of cancer and other diseases have also been noted. Furthermore, we also seek efficient diagnosis of intoxication by *i*As and overcoming of adverse effects.

## 1. Introduction

Arsenic, the 53rd most abundant element in the Earth’s crust [1], ubiquitously exists mainly as several inorganic arsenic (*i*As) species in three valence states {−3, +3, +5} and similarly for the organic compounds of arsenic (organoarsenicals, *o*As), characterised by a C-As chemical bond (Table 1 and Table 2, Appendix A). The oxidation state +1 is less environmentally significant than the others. Diverse inorganic trivalent (*i*As^(III)^, arsenite) and pentavalent (*i*As^(V)^, arsenate) species are naturally present in soils and waters [2,3,4,5,6] as solid and soluble forms depending on the Eh–pH conditions [7].

**Figure 1:**
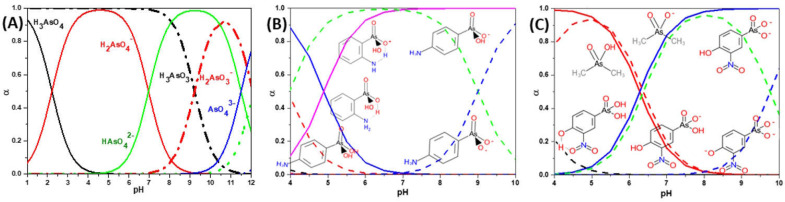
Distribution of the predominant (**A**) *i*As species, *i*As^(III)^ (dashed line) and *i*As^(V)^ (continuous line), and (**B**,**C**) some organoarsenicals. (**B**) *p*-arsanilic acid (dashed line), *o*-arsanilic acid (continuous line), and (**C**) roxarsone (dashed line), cacodylic acid (continuous line), inside the usual environmental and biological pH range.

However, under typical Eh–pH environmental and biological systems conditions, the predominant species are arsenite (H_3_AsO_3_ and H_2_AsO_3_^−^) and arsenate (H_2_AsO_4_^−^ and HAsO_4_^2−^) (Figure 1). The *o*As^(V)^ compounds typically contain the functional groups RAsO(OH)_2_ or R_2_AsO(OH) (R = alkyl or aryl), although apart from the pentaphenyl derivative As(C_6_H_5_)_5_ (Table 2), compounds of As^(V)^ with only organic ligands are rare [8]. Most important *o*As^(III)^ compounds include trimethylarsine (CH_3_)_3_As (Gosio gas) [9], dimethylarsine (CH_3_)_2_AsH, methylarsine CH_3_AsH_2_, dimethylarsine chloride (CH_3_)_2_AsCl, and methylarsine dichloride CH_3_AsCl_2_ [10]. There are also heterocycles containing As^(III)^, such as arsole, arsabenzene, and the arsenic analogues of pyrrole and pyridine. Further, arsenic-containing nucleosides (sugar derivatives or arsenosugars) exist in seaweeds [11], while some lipids can also contain arsenic [12]. Arsenicin A is the only polyarsenic compound isolated from the natural source of the New Caledonian marine sponge *Echinochalina bargibanti* [13]. The *o*As^(I)^ compounds exist in some drugs, such as Salvarsan and Neosalvarsan (Table 1), but not in natural environments. Organoarsenic compounds featuring As–Cl bonds show high toxicity, thus providing characteristic properties for the development of different chemical weapons, such as “Lewisite” (chlorovinyl-2-arsenic dichloride), “Clark I” (chlorodiphenylarsine), “Clark II” (diphenylcyanoarsine), and phenyldichloroarsine. Thus, dimethylarsine chloride (CH_3_)_2_AsCl and other chlorinated organoarsenicals were widely employed as chemical warfare agents, especially in World War I [14]. Its antidote is British anti-Lewisite (HO-CH_2_-CHSH-CH_2_SH), which alters the affinity of As^(III)^ for thiolate ligands, thereby decreasing its acute toxicity. Dimethylarsinic acid (CH_3_)_2_AsO(OH), also known as Agent Blue (cacodylic acid), was used by the US Army as an herbicide in the Vietnam War.

*i*As species are predominantly deposited in soils through natural processes from parent rocks [15] and are primarily associated with non-weathering-resistant mineral deposits, such as sulphide-rich arsenic-bearing minerals [16]. Under aerobic conditions, *i*As^(V)^ species prevail in the environment due to mineralisation processes, microbial activity, oxidation reactions, and the weathering of arsenopyrite-rich rocks [15,17]. However, *i*As are also released by natural processes, such as volcanic activity, particularly through eruptions (lava and ashes) and fumaroles (sublimates and incrustations) [15].

Rainwater solubilises some arsenic species [18], thereby transferring them to flood and storm waters, where under reducing soil conditions [19,20], As^(V)^ is quickly reduced to As^(III)^ within hours [20,21]. Nonetheless, it is usual to underestimate the geochemical mobilisation of arsenic species in anoxic systems due to the difficulty in measuring their rapid transformation and transport under low-oxygen conditions [22,23]. In aqueous environmental systems at neutral pHs in lakes, and less frequently in rivers and streams, arsenite and arsenate are usually retained on metal oxide particles (iron and aluminium), altering their distribution in environmental systems [24,25]. However, irrigation with wastewater facilitates the mobilisation of *i*As^(III)^ (and other potentially toxic metals, such as Cd, Co, Cr, Ni, Zn, Cu, and Pb) from soil particles [26], which is mainly driven by low oxygen levels dissolved in the water [27], where especially at the bottom of lakes microbial reduction is prevalent [28]. Furthermore, microorganisms can also reduce *i*As^(III)^ to arsine (AsH_3_) in soils [29]. Arsine and dimethyl arsine may be volatilised from swamps and marshy soils at pH < 6.0.

Nevertheless, agricultural, industrial, mining, medicinal, and other human activities largely contribute to environmental contamination with arsenic compounds [5,30,31]. Primary industrial uses of arsenic include the preparation of alloys, semiconductors, pigments, pesticides, glasses, and drugs, the latter employed for the treatment of diverse diseases, such as sleeping sickness (African human trypanosomiasis) and chronic myeloid leukaemia (chronic myelogenous leukaemia) [32]. Many pesticides, such as cacodylic acid, substantially enhance arsenic accumulation in soils, from where it can leach to surface and groundwater [6]. Mining techniques, such as hydraulic fracturing, mobilise *i*As into groundwater and aquifers by altering redox conditions and injecting fluids containing additional arsenic species. Many aquifers contain high concentrations of *i*As (>50 μg/L), which can lead to chronic arsenic poisoning if consumed over months or years [33]. The World Health Organisation (WHO) stipulates a limit value of 0.01 mg/L (10 μg/L) for arsenic in drinking water, since the chronic ingestion of drinking water with higher concentrations of arsenic can lead to arsenicosis [34].

Plants absorb *i*As from soil and water systems and then pass it on to animals and humans through the food chain [35,36]. Nonetheless, phytoplankton and zooplankton also play a crucial role in the bioconcentration and bioaccumulation of *i*As [37]. Consequently, *i*As accumulates in food, soil, water, and air systems, primarily affecting the human population through ingestion of contaminated food and some natural seaweeds [38]. Drinking water can contain *i*As, whereas food can contain both *o*As and *i*As species. However, *i*As species primarily abound in fruit juice and rice [39], while *o*As, such as arsenobetaine and arsenocholine, are present in marine organisms, including marine algae and certain terrestrial mushrooms. In living systems (humans, animals, and microorganisms), all *i*As species are partly methylated, forming different *o*As species, mainly monomethylarsonous acid {CH_3_As(OH)_2_, MMA^(III)^}, dimethylarsinous acid {(CH_3_)_2_As(OH), DMA^(III)^}, methylarsonic acid {CH_3_AsO(OH)_2_, MMA^(V)^}, and dimethylarsinic acid {DMA^(V)^} (Table 1 and Table 2) [40]. Rice grown in *i*As-contaminated soils can accumulate arsenic up to levels around ≈23.7 mg As/kg [41], which, depending on the amount that is ingested per day, may exceed the total arsenic, *t*As (=*i*As^(III)^ + *i*As^(V)^), dose that is ingested by humans relative to the consumption of arsenic-laced drinking water [34]. However, other countries have established limits for arsenic in food of 1 mg As/kg (seaweed and molluscs) and 2 mg As/kg (fish and crustacea), although the limit of *t*As is 1 mg As/kg for cereals (including rice) and 0.5 mg As/kg for table salts [42]. The source of arsenic in chicken is related to feed additives, such as roxarsone and nitarsone, which are widely used to control coccidiosis, a parasitic infection, and to increase the weight and skin colour of poultry [43]. However, roxarsone is no longer used in the USA, European Union, and possibly other countries as well [44].

Soluble As^(III)^ and As^(V)^ species are absorbed from the gastrointestinal tract and eliminated as *i*As^(V)^ and *o*As via the kidneys. The biological half-life of *i*As^(III)^ for human adults is about 1–3 days and slightly shorter for *i*As^(V)^ [45], established using aqueous *i*As concentrations in the range 0.15–0.58 mg/L, while other authors, using similar *i*As concentrations, stated that the half-life of *i*As^(III)^ in humans is about 10 h [46]. Consequently, additional precise information concerning this topic is still necessary. The strong relationship between urinary excretion and body retention following *i*As intake facilitates a straightforward accumulation process, with approximately 50% of the total *i*As ingested remaining in the body for a prolonged period [47]. After ingestion of *i*As, it is typically bound to thiol or sulfhydryl groups (-SH), primarily incorporated into the nails, hair, and skin and in small amounts into bones [48]. However, *i*As is primarily stored in the kidneys, liver, heart, and lungs, but in lesser amounts in muscle and neural tissue. After chronic ingestion of As^(III)^, it can be incorporated into the fingernails and toenails.

Although human exposure to *i*As can also occur through inhalation exposure [49,50], exposure to contaminated air and soil is usually minimal, but exposure to elevated concentrations of *i*As in drinking water is a significant problem in several regions worldwide [33,51]. The acute *i*As poisoning symptoms in humans begin with headaches, diarrhoea, drowsiness, convulsions, confusion, leukonychia striata (fingernail pigmentation, Mees’s, or Aldrich–Mees’s lines appear after a chronic exposure), vomiting blood, hematuria (blood in the urine), muscle cramps or spasms (charley horse), hair loss, and abdominal pain [52].

## 2. The Biological Significance of Arsenic Species

The biological significance of arsenic species is correlated with and, consequently, better understood through the mechanisms underlying the metabolism of ingested *i*As species. The chemical characterisation of any metabolic process involves elucidating and quantifying the starting metabolite, its transformation and distribution within the biological system, and potential interactions with other inorganic compounds and biomolecules, such as enzymes, cofactors, and other compounds. All metal/loid species within a biological system (whole body, organ, tissue, cell, and other constituents) relate to the **metallome**, which is synonymous with speciation [47]. On the other hand, **metallomics** (or chemical characterisation) [53,54] encompasses all analytical activities to localise, identify, and characterise qualitatively and quantitatively the chemical forms of a metal or metalloid existing in any biological system. In this way, metallomics represents a speciation analysis, which, as a first step, only reveals aspects at a particular time/point of the metabolism process. Nevertheless, the term **metabolomics** involves the complete characterisation of all metabolites, usually low-molecular-weight molecules, present in a biological system, which constitutes the **metabolome**.

We can elaborate on the above comments by simply using the term **metallome** with a specific name to refer to the arsenic species present in a biological system [55]. Thus, we use arsenome to describe the complete set of arsenic-containing species present in a given biological system, regardless of their metabolic or physiological role. At the same time, arsenobolome represents the subset of the arsenome comprising only arsenic-containing metabolites (primary metabolites) formed within the organism after the ingestion or uptake of arsenic species, including their metabolic pathways and profiles. Similarly, we introduce the term arsenobiolome to cover the subset of arsenome consisting of arsenic-containing species, other than primary metabolites, that participate directly in specific beneficial (e.g., therapeutic activity) or harmful (e.g., toxicity, carcinogenesis) processes. Consequently, **arsenomics, arsenobolomics, and arsenobiolomics** have evolved as instrumental and methodological approaches to obtain the required information at the corresponding biological level. In the following sections, we outline various aspects of the arsenic term-*omes* described above.

### 2.1. Arsenome: Speciation of Arsenic Compounds

**Figure 2:**
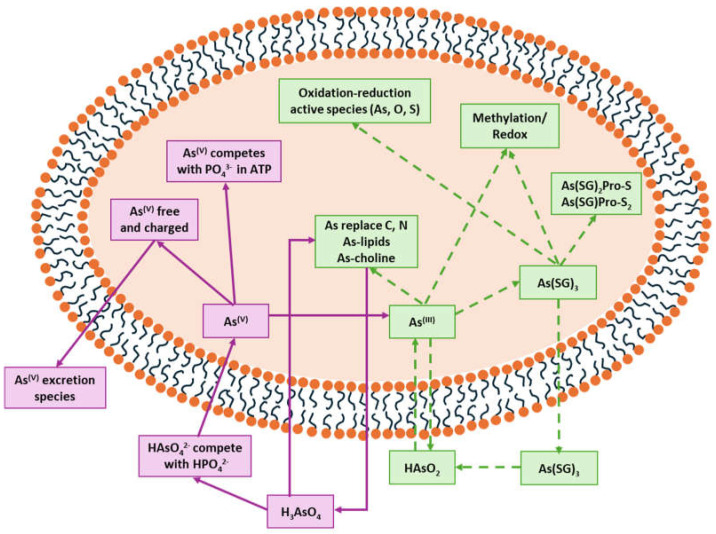
A schematic picture resuming the interrelationships and dynamic biotransformation pathways of various As^(III)^ and As^(V)^ species within the cell and in the extracellular environment. Green boxes and dashed arrows refer to As^(III)^, and purple boxes and solid arrows refer to As^(V)^. As(SG)_3_ is an important As^(III)^ species involved in the mobility and excretion of As^(III)^ into the cell. Meanwhile, As^(V)^ competes with P^(V)^ for uptake sites and ATP formation. As^(V)^ converts into As^(III)^ to facilitate elimination. As^(III)^, with the participation of AS3MT, undergoes different redox methylation.

Each arsenic species is more abundant in the organs and tissues involved in the different stages of its toxicokinetic behaviour, which depend on its biotransformation pathways during the entry, metabolism, interactions, accumulation, and excretion phases (Figure 2).

**Figure 3:**
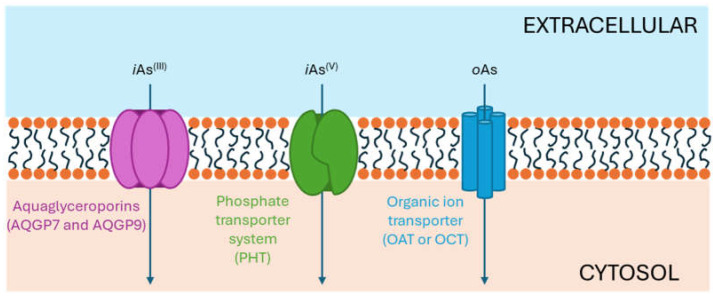
Schematic representation of the routes used by inorganic (*i*As^(III)^, *i*As^(V)^) and organic arsenic species (*o*As) to enter mammalian cells.

Both As^(III)^ and As^(V)^ are absorbed into the cell: As^(III)^ by aquaglyceroporins 7 and 9, transporting water and glycerol [56], and arsenate through a phosphate transporter system [57]. The uptake mechanisms of small molecular weight *o*As species (including arsenobetaine, arsenosugars, and arsenolipids) are largely unknown but may involve organic ion transporters (Figure 3).

Excretion of *i*As follows through multiple methylation steps [58]. Humans excrete most of the orally ingested arsenic compounds via the urine, metabolising them as mono- and dimethylated acids, and unmetabolised as *i*As^(V)^. DMA^(V)^ is typically the most abundant arsenic species in human urine after exposure to *i*As, followed by lesser amounts of MMA^(III+V)^ and *i*As species (As^(III)^ and As^(V)^). The relative proportions vary depending on individual metabolism and exposure source [59]. However, DMA^(V)^ is the primary urinary metabolite of *i*As and the major metabolite of the arsenosugars naturally occurring in seaweed [60]. Rodents and humans rapidly excrete DMA^(V)^ unchanged, together with minor amounts of trimethylarsine oxide {TMAO^(V)^, (CH_3_)_3_AsO}, a metabolite only detectable in urine at trace levels after exposure to high doses of *i*As^(III)^ and its dimethylated arsenicals (DMA^(III)^) [61]. However, most humans exposed to *i*As excrete other arsenic species, such as *i*As (20 ± 10%), MMA^(V+III)^ (15 ± 5%), and DMA^(V+III)^ (70 ± 5%) [62], although occasionally MMA^(III)^ is absent, even though this *o*As^(III)^ is an intermediary in the arsenic methylation pathway. Some particular populations excrete high amounts of MMA^(III)^ [63], particularly after oral administration of the chelating agent DIMAVAL (2,3-dimercapto-1-propane sulfonic acid) [64]. In this respect, the three trivalent arsenicals {*i*As^(III)^, MMA^(III)^, and DMA^(III)^} and the three pentavalent arsenicals {*i*As^(V)^, MMA^(V)^, and DMA^(V)^} are frequently present in human and animal urine [38,65,66,67,68], which together with other less abundant arsenic species constitute the main components of the arsenome.

Compounds containing arsenic–sulphur bonds are also subject to significant metabolic transformation in living systems, involving cleavage and reformation of these bonds during biotransformation [69,70]. Thio-arsenicals have been found in the urine, faeces, and wool of sheep after naturally consuming large doses of DMA^(V)^ [71,72,73] and arsenosugars (arsenic-containing seaweeds) [74], probably due to microbial transformations of the intestinal flora [73]. Dimethylthio-arsenicals are formed in the liver after intravenous injections of tri- and pentavalent mono- and di-methylated arsenicals [69,70], which indicates that in the presence of sulphide ions, thioarsenicals are generated in vivo in the liver and other organs. Methylated thioarsenicals, found in urine, are highly toxic, particularly dimethylmonothioarsinic acid (CH_3_)_2_AsSH or DMMTA^(V)^, which is a relevant arsenic compound in the genesis of human bladder cancer [75]. Thus, long-term exposure (of rats) to DMA^(V)^ produces thioarsenicals DMMTA^(V)^ and trimethylarsine sulphide (CH_3_)_3_AsS or TMAS in urine [72,76]. Monomethylmonothioarsonic acid (MMMTA^(V)^), DMMTA^(V)^ and DMDTA^(V)^ have also been detected in urine after exposure to *i*As or DMA^(V)^ compounds [77,78,79], which is an indication of the involvement of sulphur compounds in the metabolism of *i*As. Nonetheless, oxo-compounds are the most usual metabolites of arsenosugars excreted in the urine because thio-organoarsenates are unstable and rapidly transformed into oxo-organoarsenates [80].

**Figure 4:**
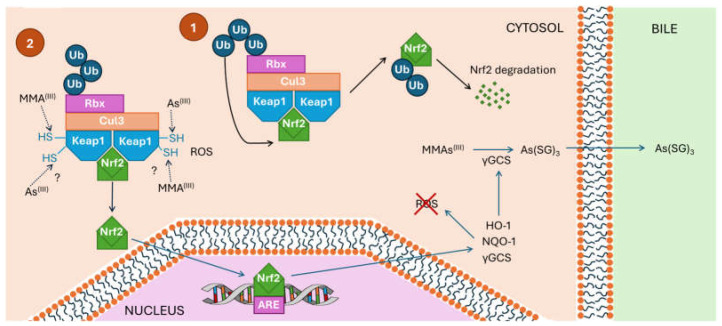
Mechanism triggering the biological transformation of As^(III)^ (*i*As^(III)^ and *o*As^(III)^, exemplified by MMA^(III)^), for its excretion from the cell into the bile. Route 1 refers to the basal situation, where Nrf2 suffers ubiquitination via Keap1-Cul3 ubiquitin ligase complex and it is degraded into the proteosome. Route 2 refers to oxidative stress situation: MMA^(III)^ and *i*As^(III)^ can interact with the Keap1 protein releasing Nrf2, which triggers ARE and EpRE response, increasing antioxidant proteins as consequence which results in As^(III)^ excretion as As(SG)_3_ into the bile. The interaction mechanism between arsenic and Keap1 is still unclear.

The biotransformation of *i*As for excretion occurs through the activation of the nuclear factor erythroid 2-related factor-2 (Nrf2), inducing a beneficial antioxidant response but also a cell-damaging effect [81,82] (Figure 4). However, binding of Nrf2 to Kelch-like ECH-associated protein 1 (Keap1) in its inactive form counteracts the detrimental effects raised by *i*As [83]. Keap1, through its thiol or sulphydryl (-SH) moieties, binds electrophilic arsenic species, such as MMA^(III)^, and induces the release of Nrf2, migrating through the cytoplasm to the nucleus [81]. MMA^(III)^ does not directly engage with Nrf2 but instead modulates Keap1 through post-translational modifications. MMA^(III)^ and *i*As^(III)^ interact with the Keap1 protein by disrupting the Keap1-Cul3 E3 ubiquitin ligase complex, thereby preventing Nrf2 degradation and leading to its accumulation and subsequent activation of the Nrf2-antioxidant response pathway [84]. Many other post-translational modifications that activate the Nrf2 pathway to regulate antioxidant and anti-inflammatory responses are under investigation, although the modification sites differ, being typically at Cys151 in Keap2. The protein Nrf2 activates the antioxidant responsive element (ARE) and electrophile responsive element (EpRE), which contribute to increasing antioxidant proteins [85], where heme oxygenase 1 (HO-1), NAD(P)H-quinone oxidoreductase 1 (NQO-1), and γ-glutamylcysteine synthase (γGCS) work in combination to reduce the oxidative stress (OS). It seems that the presence of γGCS facilitates liberation of arsenite triglutathionine As(SG)_3_ from the liver into the bile for excretion, thus removing As^(III)^ from the cell [81].

In humans, *i*As species accumulate in diverse tissues and body fluids (bone marrow, cardiac, central nervous system, blood, gastrointestinal, gonadal, kidney, liver, pancreatic, and skin) [86,87]. The chemical structure of the *i*As^(V)^ ion resembles that of phosphate, thus replacing it in bones. However, in mammal liver, *i*As is enzymatically and non-enzymatically methylated, generating DMA^(V)^, which is excreted (greater than 90%) in the urine [87]. Nonetheless, As^(III)^ can bind to proteins in red blood cells (RBCs) [88], which are involved in the regulation of gene expression. This interaction may, over prolonged exposure, lead to the molecular changes associated with certain cancers (including skin, bladder, kidney, liver, lung, and prostate), although the precise mechanisms remain under investigation [87].

### 2.2. Arsenobolome: Primary Metabolites of Ingested Arsenic-Containing Compounds

The term arsenobolome refers to the arsenic metabolome’s equivalent content (metabolic dynamics, arsenic profiles). A good understanding of the biochemical processes involved in the metabolism of arsenic compounds would enable us to selectively inhibit (or reduce) the chemical mechanisms that give rise to the most toxicological forms of arsenic or, at the very least, induce an appropriate metabolic pathway to generate less toxic arsenic species. Understanding arsenic-based metabolites is crucial for comprehending their metabolic processes, body burden, and associated health effects.

**Figure 5:**
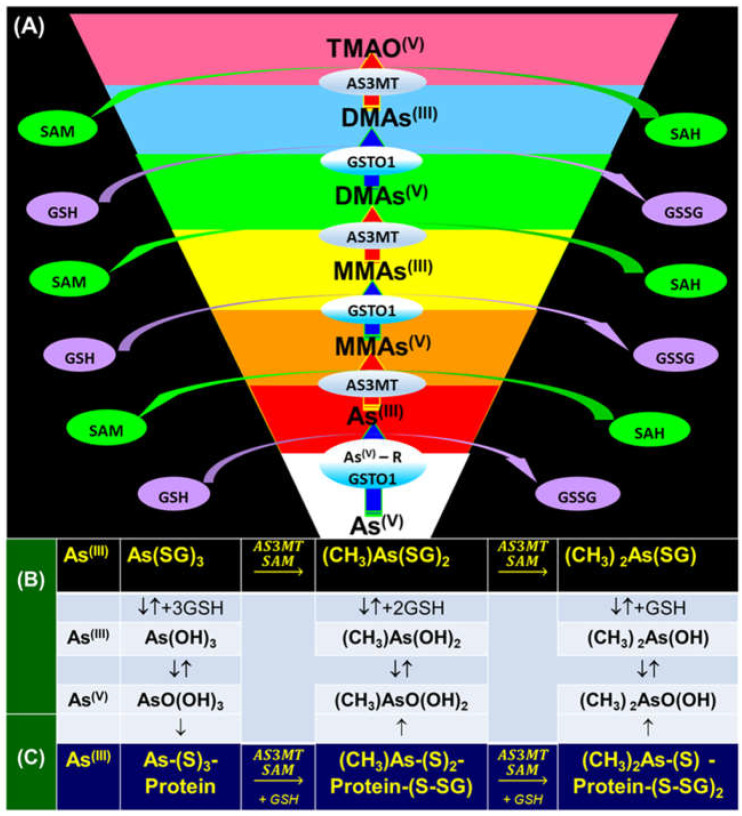
A pictorial schematic representation of the (**A**) conventional oxidative arsenic methylation pathway and (**B**,**C**) reductive arsenic methylation pathways forming As(GS)_x_ complexes (**B**) and As-protein complexes (**C**) in humans. The arrows indicate the direction of the chemical transformation. GSTO1 (Glutathione S-Transferase superfamily, Omega-1), GSH (Glutathione), GSSG (Oxidised glutathione), AS3MT (Arsenite methyltransferase is an enzyme encoded in humans by the *AS3MT* gene), SAM (S-adenosylmethionine), SAH (S-adenosyl-L-homocysteine). Other symbols appear throughout the text. Corel^®^ Photo-Paint software, version 24.3.0.571.

Inside the cell, *i*As^(III)^ participates in different redox and substitution reactions, forming oxo-arsenical species and binding to the –SH groups of proteins. The *i*As^(III)^ species show high affinity for vicinal –SH groups in proteins, such as the disulphide oxidoreductase family-like glutathione reductase [89] and thioredoxin reductase (TR), thereby inhibiting their activity. In this way, the bacterial strain ULPAs1 (β-Proteobacteria), together with α- and β-Proteobacteria, oxidise *i*As^(III)^ to *i*As^(V)^ [90], which presumably evolved as a detoxification process and energy generation. On the other hand, *i*As^(V)^ can also be reduced by glutathione (GSH) to *i*As^(III)^ [91]. Thus, the enzyme purine nucleoside phosphorylase (PNP) (enzyme encoded in humans by the *NP* gene), bearing a dithiol group, reduces *i*As^(V)^ in vitro to *i*As^(III)^ [81,92,93], although it is unknown if this reduction also unfolds in vivo [94]. The reduction in iAs^(V)^ to *i*As^(III)^ is an energy-generating process for microorganisms, serving as a necessary step before *i*As^(III)^ methylation. Consequently, the detoxification of *i*As^(III)^ occurs through complex methylation and redox processes [95] (Figure 5). In mammals, *i*As^(III)^ methylation primarily occurs in the liver, with smaller quantities being methylated in the kidney and lung [96].

The methylation mechanism of *i*As in humans remains unresolved, although methionine is the source of methyl groups, and arsenic methyltransferase (AS3MT, Cyt19) is the enzyme involved [10,97]. In many mammals, including humans, all AS3MTs utilise S-adenosyl-methionine (SAM, AdoMet) as a cofactor [98], with As^(III)^ as the preferred substrate. Consequently, the most probable mechanism of in vivo *i*As^(V)^ methylation would involve a previous 2-electron reduction in As^(V)^ to As^(III)^ to fulfil the subsequent methyl transfer reaction [64,99] (Figure 5). This behaviour behind the methylation of As^(III)^ by AS3MT is a spontaneous exergonic ≈38 kJ/mol process under standard conditions [100]. All As^(III)^ species are more toxic than As^(V)^ species, and more readily methylated [63]. The principal methylation pathway depends on the cell type [99], resulting in the formation of MMA^(III)^ and DMA^(III)^ metabolites. As a whole, we can view the enzymatic methylation of *i*As to MMA^(III)^, DMA^(III)^ or trimethylarsine (TMA^(III)^) as a detoxification process if the methylated As^(III)^ intermediate is rapidly excreted via urine [101], but the reduction in As^(V)^ to As^(III)^ might be considered a bioactivation, as As^(III)^ is more toxic than As^(V)^ [62]. Methylation can be viewed as a toxification process [48,102,103] because the mono-methylated compounds are more reactive and toxic than the *i*As compounds to human hepatocyte, keratinocyte, and bronchial epithelial cells [104,105]. However, although *i*As and methylated As^(III)^ metabolites cross the placenta to the foetus, the maternal methylation capacity for *i*As increases during pregnancy, particularly in the first trimester and independent of folate status [106], leading to enhanced urinary excretion of mono- and dimethylated arsenic species. Current evidence indicates that this increased methylation occurs primarily in the mother, which may help reduce foetal exposure [63,107].

The enzymatically catalysed methylation of *i*As (Figure 5) by AS3MT can occur via two primary routes (Figure 5A,B) [108,109]. However, a third route, similar to the second one (Figure 5B), but involving the participation of proteins, particularly as a substrate, is also accepted (Figure 5C) [110]. The third route considers that only As^(III)^ metabolites bind to high-molecular-weight proteins, and *i*As is methylated, followed by a reduction process if glutathione (GSH) is present. However, it does not follow stepwise oxidative methylation with MMA^(V)^ and DMA^(V)^, which are only present as final metabolites [111].

**Figure 6:**
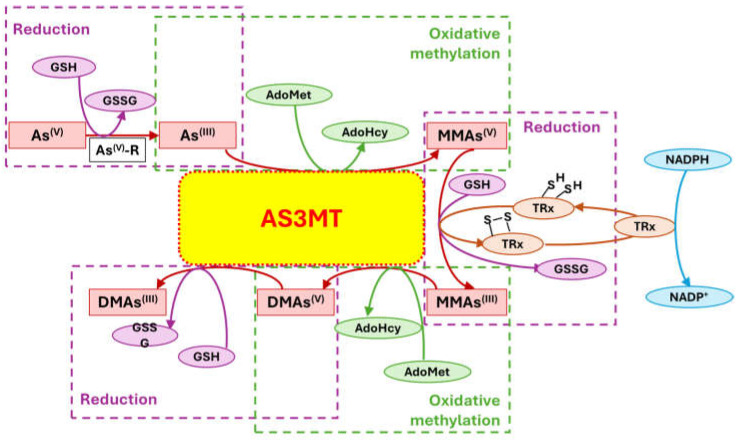
Diagram showing the arsenic biotransformation processes catalysed by As^(III)^ methyltransferases (AS3MT), which facilitate the stepwise methylation of trivalent arsenic species, contributing to their detoxification and subsequent excretion.

The first route (Reaction (1)) outlines an enzymatic oxidative methylation process of *i*As^(III)^ and MMA^(III)^ in cells of different organisms (humans, rats, fish, and bacteria, among others), which produces MMA^(V)^ and DMA^(V)^ [40], according to the following sequence (Figure 5A and Figure 6),

(1) $${iAs}^{\left(III\right)}\overset{+e^{-},+{Me}^{+}}{↔}{MMA}^{\left(V\right)}{\overset{-2e^{-}}{↔}MMA}^{\left(III\right)}\overset{+e^{-},+{Me}^{+}}{↔}{DMA}^{\left(V\right)}\overset{-{2e}^{-}}{↔}{DMA}^{\left(III\right)}$$

The enzyme AS3MT transfers a methyl group from the co-factor SAM to *i*As^(III)^, generating a mono-methylated As^(V)^ compound, MMA^(V)^ [81], and S-adenosyl-L-homocysteine (SAH).

Using a multimodular protein, CmArsM, a 400-residue thermostable enzyme with 44,980 Da, orthologue of AS3MT, the reaction mechanism between *i*As^(III)^ and SAM provides the interaction of *i*As^(III)^ with the thiol groups of the cysteine (Cys) residues in Cys72, Cys174, and Cys224. In this process, the SAM sulphur atom carries a positive charge, which attracts an electron from the carbon atom to bond to the methyl group, facilitating a bimolecular nucleophilic substitution (SN_2_) reaction with the lone pair of electrons on the arsenic atom to form an As–C bond, leaving SAH [112].

The MMA^(V)^ formed in Reaction (1) acts as a precursor to DMA^(V)^. However, MMA^(V)^ needs a previous reduction to MMA^(III)^ by methylarsonate reductase, a member of the Glutathione S-Transferase superfamily, omega-1 (GSTO1), or omega-2 (GSTO2) [81], which are essential rate-limiting enzymes of *i*As biotransformation [113]. The MMA^(III)^ is methylated again by AS3MT through the co-factor SAM to form DMA^(V)^ [114], SAM + CH_3_As(OH)_2_ ↔ SAH + DMA^(V)^, which can be converted to DMA^(III)^ by GTSO1 [81]. However, the cytosolic concentration of SAM varies among tissues; typically, in the sequence, testis > kidney > lung > liver [115,116].

The second route (Reaction (2)) involves thiol-containing *i*As^(III)^ complexes, in which methylation occurs by a non-oxidative mechanism. In this route, *i*As^(III)^ species acquire two methyl groups first, making a complex with the thiol-containing tripeptide GSH (enzyme encoded in humans by the *GSR* gene) and probably also lipoic acid (6,8-dithiooctanoic acid),

(2) $${iAs}^{\left(III\right)}\overset{+{SH}^{-}}{↔}{{As}^{\left(III\right)}\left(SG\right)}_{3}{\overset{+2e^{-},+{Me}^{+}}{↔}MM{As}^{\left(III\right)}\left(SG\right)}_{2}\overset{+{Me}^{+}}{↔}DM{As}^{\left(III\right)}\left(SH\right)$$

In this second route, the metabolism of *i*As^(III)^ to form methylated arsenicals occurs by AS3MT, transferring a methyl group from SAM to the As^(III)^ centre, but only when GSH is present via arsenic triglutatione (ATG^(III)^, As^(III)^(SG)_3_), which transforms into monomethylarsino diglutathione (MMADG^(III)^ or MMA^(III)^(SG)_2_) [81]. Furthermore, AS3MT can methylate this complex again, establishing equilibrium with the DMA^(III)^-SG (DMAG^(III)^, dimethylarsino glutathione) [81,117]. Both MMADG^(III)^ and DMAG^(III)^ are unstable at low GSH concentrations (2.4 mmol/L), hydrolysing and oxidising to MMA^(V)^ and DMA^(V)^, respectively. This model requires the scission of an As^(III)^–S bond between the cysteinyl residue of GSH and As^(III)^. The *o*As^(V)^ compounds have an affinity to thiol groups lower than *i*As^(V)^ ones [99], but GSH can reduce non-enzymatically *o*As^(V)^ [94]. Thus, GSH or glutathione-S-transferase omega class 1-1 (GSTO1-1) (enzyme encoded by the *GSTO1* gene) reduces MMA^(V)^ to MMA^(III)^ [118]. In a second reaction, MMA^(III)^ can be oxidatively methylated to DMA^(V)^ [119], while some DMA^(V)^ can also be reduced to DMA^(III)^ (Figure 5B). The *GSTO1* gene is identical to the *AS3MT* gene [120], but the latter uses thioredoxin (Trx) and NADPH as a reducing agent, whereas GSTO1-1 uses GSH. The activity of AS3MT (methylation process) increased with the presence of GSH and, notably, if the reductants Trx/TR/NADPH-coupled system is present. However, the omission of Trx sharply reduces activity with minimal formation of MMA, suggesting some effect of GSH on AS3MT’s catalytic activity. TMAO^(V)^ is present in reactions containing AS3MT, SAM, *i*As^(III)^, and TCEP (tris(2-carboxyethyl)phosphine) but not GSH.

On the other hand, the proposed metabolism of DMA^(V)^ includes (i) the first reduction in DMA^(V)^ to DMA^(III)^, a more toxic and genotoxic compound [121], which then transforms into DMMTA^(V)^ and DMMTA^(III)^; (ii) formation of TMAO^(V)^; and (iii) reduction in DMA^(V)^ to (CH_3_)_2_AsH, which transforms to peroxyl (^•^O-O-R), hydroperoxyl (H-O-O^•^), hydroxyl (^•^O-H), and superoxide (O_2_^•^) radicals [113,122].

**Figure 7:**
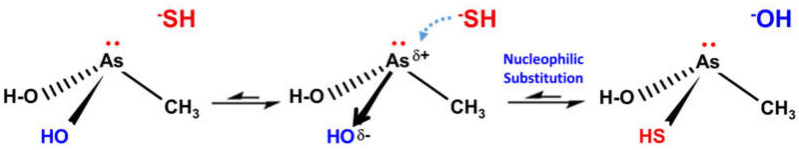
Replacing an arsenic–oxygen (As–OH) group with an arsenic–sulphur (As–SH) group is indeed an oxidation reaction, specifically a nucleophilic substitution where the reaction involves the sulphur atom acting as a nucleophile, attacking the arsenic centre and displacing the oxygen.

Some thioarsenicals, DMMTA^(V)^, and smaller amounts of DMMTA^(III)^, appeared as metabolites in sheep’s urine, which naturally consumed arsenosugars from seaweed as a primary food source [69,70]. Further, incubation of MMA^(III)^, DMA^(V)^, and DMA^(III)^ in rats, hamsters, mice, and human RBCs, measuring the uptake rates and the arsenic species present, has confirmed that DMA^(III)^ is taken up more efficiently than DMA^(V)^ [88]. DMA^(III)^ transforms in the presence of sulphide ions to DMMTA^(III)^ and DMMTA^(V)^ by oxidative replacement of the (As-)OH group (Figure 7) with the (As-)SH group [74,123,124]. The As-S bond in MMADG^(III)^ and DMAG^(III)^ is more stable than in ATG^(III)^, suggesting that the stability of the As–S bond is critical in the disposition/excretion of methylated As^(III)^ compounds in mammals [124]. GSH-conjugated forms of trivalent arsenicals, ATG^(III)^ and MMADG^(III)^, but not DMAG^(III)^, have been detected in bile [125,126,127]. DMAG^(III)^ constitutes a key metabolite in the metabolism of *i*As, which is seemingly excreted into the bloodstream in a trivalent form, although the underlying mechanism is not yet understood [111,117]. The chemical kinetics and potency of methylated thioarsenicals and oxyarsenicals As^(V)^ differ when acting as cytotoxicants and genotoxicants [128]. So, the formation, fate, and effects of methylated thioarsenicals require bridging the molecular processes underlying methylation and thiolation reactions [128].

The methylation rate of As^(III)^ varies among species and ethnicity [129], with essential differences between individuals, including functional genetic polymorphisms, age, sex, pregnancy, body weight, arsenic species, dose levels, exposure routes, smoking habits, nutritional status, and dietary composition affecting the different susceptibility to *i*As-induced health hazards [64,130,131,132]. Thus, women, particularly during pregnancy, have greater methylation capacity than men and exhibit increased *t*As levels in urine [133]. Similar enhanced methylation of As^(III)^ occurs in children, but not in adults [134]. Furthermore, smoking and alcohol consumption relate to a low As^(III)^ methylation capacity, while subjects with skin lesions show a low arsenic methylation capacity [135]. A robust genetic association exists between three polymorphic sites in the *AS3MT* gene and the DMA^(V)^ to MMA^(V)^ ratio in Mexican children (7–11 years old) but not in adults (18–79 years old) [64]. Thus, single-nucleotide polymorphisms in GSTO1, GSTO2, and PNP (purine nucleotide phosphorylase) are associated with As-induced skin lesions, which are the most common signs of *i*As toxicity [136]. The strong association between AS3MT and GST polymorphisms, that affect *i*As metabolism, has significant implications for the toxicology and pharmacogenetics of As^(III)^ with substantial repercussions for public health and government regulations [137,138].

Many organisms methylate *i*As, but some mammals, including the guinea pig, marmoset monkey, chimpanzee, and many South American monkeys, are incapable of methylating *i*As because they are deficient in or have slightly different forms (genetic or ethnic polymorphism) of AS3MT [64]. Alternatively, nutritional status also plays a crucial role in arsenic methylation, where folate may facilitate the methylation and excretion of As^(III)^ [134], linking As^(III)^ metabolism with one-carbon/folate metabolism and histidine catabolism. For this reason, it is unclear whether diabetes is related to arsenic metabolism or is a consequence of the metabolism of one-carbon molecules [139]. A protein-altering variant in FTCD (formiminotransferase cyclodeaminase) increases urinary excretion of *i*As and MMA^(III)^ but not DMA^(III)^, also inducing skin lesions [140,141]. Other detoxification pathways are activated by methylenetetrahydrofolate reductase and glutathione S-transferase, which are involved in *i*As^(III)^ metabolism in humans, and polymorphisms in the genes that encode these enzymes play a role in susceptibility to arsenic-induced cancer [142], which can be influenced by environmental, genetic, and nutritional factors.

Low levels of other *o*As, such as arsenobetaine {(CH_3_)_3_As^+^CH_2_COO^−^, AsBet} and arsenocholine {(CH_3_)_3_As^+^CH_2_CH_2_OH, AsCho}, are also present in living systems, although AsCho is oxidised to AsBet in vivo, ubiquitously present in marine and terrestrial habitats [143]. Nonetheless, the biosynthesis of AsBet also occurs from dimethyl arsinoyl ethanol {(CH_3_)_2_As(O)CH_2_CH_2_OH}. This process involves an oxidation stage, generating the acidic carboxymethyl group {-CH_2_COOH} of dimethyl arsinoyl acetate {(CH_3_)_2_As(O)CH_2_COO^−^}, catalysed by a methyltransferase enzyme using SAM as a methyl donor [144]. The reversible action of a methyltransferase also catalyses the bioconversion of AsBet to (CH_3_)_2_As(O)CH_2_COO^−^ [145].

### 2.3. Arsenobiolome: Arsenic Species with Specific Beneficial or Harmful Activity

Arsenobiolome refers to the different forms of arsenic and their beneficial and harmful effects.

#### 2.3.1. General Toxicological Effects of Arsenic Species

As^(III)^ is highly toxic and a carcinogen (classified as Group I by the International Agency for Research on Cancer, IARC) [146]. Strongly associated *i*As toxicity problems mainly include cardiac, respiratory, gastrointestinal, reproductive system, liver, kidney, nervous, and neurological disorders [10], developing especially skin, lung, and bladder cancers, disabling the immune system, and generating coma and death [147,148]. Additionally, *i*As causes dermatological manifestations, such as raindrop pigmentation and hyperkeratosis, also facilitating the excretion of several compounds in the urine: (i) uroporphyrin (2,7,12,17-porphine-tetra propionic acid, any of the four isomeric porphyrins) (uroporphyrinuria), (ii) coproporphyrin (3,8,13,17-tetramethylporphyrin-2,7,12,18-tetrapropanoic acid or any related porphyrin compounds originated from the decomposition of bilirubin and haemoglobin) (coproporphyrinuria), and (iii) protein (tubular proteinuria). Furthermore, total blood arsenic concentrations are associated with diverse metabolic alterations, including those directly related to one-carbon metabolism (OCM) pathways [149]. A relationship exists between human exposure to *i*As and diverse diseases, and further studies are needed to confirm this causal association [150].

Even though conditioned by its clearance rate from the body, *i*As^(III)^ species are more toxic than *i*As^(V)^ [151]. The gas arsine, AsH_3_, also shows a strong toxicity [152]. Nonetheless, *o*As^(V)^ species are less toxic than *i*As^(V)^ [9]. This way, methylation is classically the primary mechanism of biotransformation and detoxification for *i*As [153]. However, methylated As^(III)^ species are, in general, more toxic than *i*As^(III)^ to humans and animals [105,154], although they are excreted more rapidly in urine [91], thereby addressing the potential drawback generated through methylation. The oral LD_50_ values (for rats) differ broadly for *i*As^(V)^ (100 mg/kg), *i*As^(III)^ (41 mg/kg), MMA^(V)^ (961 mg/kg), and DMA^(V)^ (644 mg/kg) [155], although the LC_50_ value found in Chang human hepatocytes using tetrazolium salt is 13.6 μmol/L for MMA^(III)^ versus 164 μmol/L for *i*As^(III)^ [104].

The most toxic arsenic compound, AsH_3_ [152], causes haemolysis of RBCs, leading to haemolytic anaemia, which is responsible for the development of oliguria (diuresis), a primary criterion for diagnosing acute kidney injury (AKI) (or acute renal failure). AsH_3_ can also interact with the -SH groups of proteins and enzymes, inhibiting the erythrocyte sodium–potassium pump [156].

Several *i*As^(III)^ compounds, such as NaAsO_2_ and As_2_O_3_, cause oxidative stress (OS), genotoxicity, cytotoxicity, cell cycle arrest, and apoptosis. However, the use of As_2_O_3_ improves production of reactive oxygen species (ROS), significantly decreasing superoxide dismutase (SOD) activity and intracellular GSH levels compared with NaAsO_2_ [157]. Furthermore, DNA damage, chromosomal breakage, cell cycle arrest, and apoptosis are more severe in the As_2_O_3_-treated cells than in the NaAsO_2_-treated cells at the same *t*As content [158]. This paradoxical differential behaviour of NaAsO_2_ and As_2_O_3_ results in distinct mechanisms of carcinogenesis and anticancer function [159], likely due to minimal differences in the chemical structure of the same *i*As^(III)^ solutions, as suggested by Raman spectroscopy [7]. These differences in the behaviour of both compounds, even though both compounds provide *i*As^(III)^ ions in solution, are still unclear. Their different biological behaviour is probably due to differences in the pH of the solutions prepared with both compounds. It is assumed that the arsenic species As(OH)_3_ is the most stable structure existing in solution at neutral pH. However, at neutral pH, As(OH)_3_ and AsO(OH)_2_^−^ coexist at different concentrations [160,161], which, together with the fact that As_2_O_3_ provides twice the number of *i*As^(III)^ ions per molecule, means that the arsenic solutions injected into the experimental subjects can be quite different.

*i*As^(III)^ and its methylated derivatives, MMA^(III)^ and DMA^(III)^, are more toxic than the corresponding pentavalent arsenicals, *i*As^(V)^, MMA^(V)^, and DMA^(V)^ [162,163]. The toxicity of *i*As^(III)^ is associated with the generation of ROS within cells [164,165,166] and its effective interactions with the –SH groups of proteins [167,168]. Consequently, *i*As^(III)^ species inhibit many enzymes [169], such as succinate dehydrogenase and pyruvate dehydrogenase (PDH) [169], thereby elevating lactate dehydrogenase levels and leading to lactic acidosis [170]. Furthermore, *i*As^(III)^ species can inhibit DNA repair processes [171], thereby enhancing cancer susceptibility [172], and causing other multi-system non-cancerous effects [2,173]. In rats, *i*As^(III)^ enhances in vitro arterial thrombosis, increases serotonin levels, thromboxane A2 formation, and platelet adhesion proteins in platelets (similar to human platelets) [174]. Human exposure to *i*As can induce atherosclerosis by increasing platelet aggregation and reducing fibrinolysis, leading to arrhythmia and increased cellular calcium overload [175]. In addition, *i*As upregulates the expression of interleukin-1, tumour necrosis factor-α, vascular cell adhesion molecule, and vascular endothelial growth factor, thereby inducing cardiovascular pathogenesis [176].

Like *i*As^(III)^ and unlike MMA^(V)^, MMA^(III)^ binds strongly to the SH-group of proteins. As a result, MMA^(III)^ and DMA^(III)^ are in vitro and in vivo significantly more cytotoxic to human and animal cells than *i*As^(III)^, assuming the decreasing toxicity sequence MMA^(III)^ > DMA^(III)^ > *i*As^(III)^ > *i*As^(V)^ > MMA^(V)^ ≥ DMA^(V)^ [105,119,154,162,164,177,178,179]. MMA^(III)^ species are highly reactive, being a much more potent inhibitor than *i*As^(III)^ and MMA^(V)^ for glutathione reductase, glutathione peroxidase [180], PDH [177], and especially TR [181,182]. Conclusively, methylation is not solely involved in a detoxification mechanism of *i*As^(III)^ but could also represent a pathway for its activation [183]. We can note the greater toxicity of methylated As^(III)^ with respect to genotoxic effects, particularly in humans, who exhibit higher methylation rates than those excreting less methylated species and, consequently, more *i*As [184]. Therefore, MMA^(III)^ must be considered a pivotal intermediary to understand the genesis and evolution of *i*As carcinogenicity.

The high toxicity of the trivalent and pentavalent thioarsenicals warrants additional research, as amino acids (Cys) and peptides (GSH) containing –SH groups bind As^(III)^ metabolites more effectively than those of As^(V)^ [183]. Thus, the cytotoxicity of DMMTA^(V)^ (LC_50_ 10.7 μmol/L) is higher than that of *i*As^(III)^ (LC_50_ 5.5 μmol/L) and DMA^(III)^ (LC_50_ 2.2 μmol/L) for cultured A431 human epidermoid carcinoma cells [185,186,187,188]. This high toxicity is also relevant to *i*As-induced carcinogenicity in the urinary bladder [75], whilst EJ-1 human bladder cancer cells follow the decreasing toxicity sequence DMA^(III)^, DMMTA^(V)^ > *i*As^(III)^ ≫ *i*As^(V)^ > MMMTA^(V)^ > MMA^(V)^, DMA^(V)^, and DMDTA^(V)^. Exposure of EJ-1 human bladder cancer cells to DMMTA^(V)^ reduces the expression of the p21 and p53, accompanied by increased DNA damage and elevated intracellular ROS levels. However, *i*As^(III)^ significantly increases the protein expression of p21 and p53, as well as the intracellular GSH contents in human bladder cancer EJ-1 cells, but not the ROS levels at the IC_50_, although ROS levels increase after prolonged exposure to *i*As^(III)^ [75]. Exposure of human bladder cancer EJ-1 cells to DMA^(III)^ or DMMTA^(V)^ reduces the GSH levels, suggesting that DMMTA^(V)^ causes cell death through OS [75].

Arsenic (arsenite) also acts as an antagonist to selenium (Se) as selenite in vivo through the formation of seleno-bis (S-glutathionyl) arsinium ion, [AsSe(GS)_2_]^−^ [189,190], accelerating its excretion from the bile [191] and affecting its metabolism in vivo [192]. Thus, Se deficiency can result from chronic exposure to *i*As [161,193]. In particular, high concentrations of *i*Se^(IV)^ in serum (0.160–0.174 μg/g creatinine) correlate with reduced methyl and dimethyl arsenic levels in urine in human studies [194]. In other words, the concurrent exposure to *i*Se^(IV)^ and *i*As^(III)^ inhibits methylation. It increases retention of *i*As^(III)^ [195], which is significantly higher in tissues and, consequently, has more pronounced toxic effects [102], also forming [AsSe(GS)_2_]^−^ in liver tissues, which is then rapidly excreted via bile into the intestinal tract [193]. Se^(IV)^ and N-acetylcysteine (NAC) play a similar role, protecting against As_2_O_3_-induced cytotoxicity, DNA damage, and apoptosis in PLHC-1 cells [196]. On the other hand, other interacting compounds involve the vitamin B complex, where the intake of thiamin (B1), niacin (B3), pantothenic acid (B5), and pyridoxine (B6) correlates with an increased *t*As excretion in humans [196].

Many microorganisms from As^(III)^-enriched environments are resistant to *i*As toxicity [151,197,198,199,200]. The resistance of *Escherichia coli* to *i*As links to the combined effect of the following five genes [201,202]: (i) ArsR/metal-binding protein with a domain for *i*As^(III)^ (and *i*Sb^(III)^ as well); (ii) ArsD/metal-binding protein with three pairs of vicinal Cys residues; (iii) ArsA, a catalytic unit of arsenite-translocating ATPase; (iv) ArsB membrane sector of ArsA gene product; and (v) ArsC, an arsenate reductase. However, similar studies need to be conducted on mammalian cells. The resistance involves reduced uptake of *i*As^(V)^ and increased phosphate (PO_4_^3−^) transport within the bacterial cell, resulting from a possible intracellular competition between *i*As^(V)^ (AsO_4_^3−^) and PO_4_^3−^ ions. Certain microorganisms can develop alternative resistance mechanisms to *i*As toxicity by reducing the less toxic *i*As^(V)^ form to the more toxic *i*As^(III)^, which is then more rapidly excreted [9,203,204]. The OS adaptation factors, such as ABC transporter proteins (ABCC1, ABCC2), GST-π, and SOD, together with efflux As^(III)^, heme oxygenase-1 (HO-1), and Nrf2, usually facilitate As^(III)^ tolerance, playing essential roles in innate resistance to As^(III)^ [205].

From a general perspective, *i*As intoxication in mammals can occur at high and low doses and over short and chronic exposure times. Intoxication by high concentrations of *i*As^(III)^ results in its accumulation in cells, with effects quite different from those observed with a slow accumulation of low As^(III)^ doses. The latter situation would allow tolerance mechanisms to come into play. Low doses (μg/L concentrations in drinking water) of *i*As^(III)^ species show aneugenicity (activity to induce aneuploidy or an abnormal number of chromosomes) [206]. However, at high doses (μg/L concentrations in drinking water) of As^(III)^, it behaves as a clastogenic agent causing disruption or breakages in chromosomes in human keratinocytes [207,208]. The human adults’ minimal lethal dose for acute As^(III)^ poisoning is around 100–300 mg [209] or ≈0.6 mg/kg/day [210], although the toxicity level also depends on the chemical form present. Nonetheless, differences in the acute and chronic effects of As^(III)^ remain poorly understood, particularly in terms of signal transduction. Thus, after acute (24 h) exposure (human keratinocyte and fibroblast cells) to As^(III)^, the generation of ROS activates AP-1 (activator protein 1) and NF-κB (nuclear factor-κB), resulting in c-fos/c-jun activation, over-secretion of pro-inflammatory and growth-promoting cytokines stimulating cell proliferation [211,212,213,214]. In contrast, long-term exposure (10–12 weeks) of human fibroblast cells to *i*As^(III)^ induces carcinogenesis [215], via several mechanisms, including genotoxicity, altered cell proliferation, OS, epigenetic changes, signal transduction disorders, cytotoxicity, and regenerative proliferation [216].

Early-life exposure to the following doses of *i*As in drinking water, 2 weeks before pregnancy until the birth of the F1 offspring, 1, 10, 245, and 2300 μg/L, induces phenotypic changes in offspring and subsequent generations of foetal primordial germ cells (PGCs), influencing the long-term health of the rat lineage [217]. Changes in body weight in a rat model with chronic exposure to arsenic in drinking water (1 mg As_2_O_3_/L) have been shown to decrease sperm quality, likely due to DNA methylation in ovaries and testes, DNA damage in the white blood cells (WBC), and aberrant gonad morphology [218]. Notwithstanding, there are dose-dependent effects of As^(III)^ exposure in utero in humans, including genetic damages in newborns as determined by 8-OHdG, 8-nitroguanine, DNA strand breaks, and micronuclei (MN) frequency, which facilitate the later development of various diseases, including cancer [219]. Furthermore, DNA-N7-methylguanine (DNA-N7-MeG) in urine is an indicator of alkylating agent exposure in humans, and As^(III)^-induced N7-MeG levels in urine of pregnant women significantly associated with an increased risk of adverse birth outcomes in newborns, emphasising maternal N7-MeG as a sensitive and effective biomarker for newborn health, particularly after early-life As^(III)^ prenatal exposure to low-to-moderate levels of arsenic [220].

In the following subsections, we highlight some particular toxicological effects of arsenic species.

##### Signal Transduction

As^(III)^ alters different cellular signalling, inducing ROS, neoplastic transformation, carcinogenesis, and apoptosis, also affecting different the cell cycle, growth factors, mitogen-activated protein kinase pathways, NF-ĸβ regulation, cellular proliferation, and matrix-metalloproteinases (MMPs) [221]. Human exposure to *i*As affects signal transduction, inducing heat shock proteins (HSP) [211,222] and increasing OS by generating ROS and reactive nitrogen species (RNS) [22], with ROS playing the most crucial role in exerting arsenic’s toxic effects [223]. Moreover, *i*As generate OS by depleting the cell’s antioxidants containing –SH groups [48]. Oxidative stress causes cell damage, altering glucose homeostasis and lipid metabolism, which facilitates the development of metabolic syndrome X, obesity, insulin resistance, inflammation, and type 2 diabetes [224,225,226]. Oxidative stress disrupts the signal transduction pathways of the nuclear transcriptional factors PPARs (peroxisome proliferator-activated receptors) [227], which play a crucial physiological role in the ω-hydroxylation of fatty acids. Further, the OS also inhibits pro-inflammatory transcription factors (the nuclear factor kappa beta, NF-κB, and the activator protein-1, AP-1), pro-inflammatory cytokines (interleukins, IL-1, -6, -8, -12, and tumour necrosis factor, TNF-α), pro-inflammatory nitric oxide (NO), inducible nitric oxide synthase (NOS), cell adhesion molecules (ICAM-1 and VCAM-1), and anti-apoptotic factors [87,215,224,226,228,229,230,231,232,233,234]. The OS inhibits antioxidant enzymes, such as SOD and catalase, but particularly GSH-dependent enzymes, like glutathione-S-transferases (GSTs), GSH peroxidase (GSH-Px), GSH reductase [179], and TR [181], via binding to the –SH groups [235,236]. The last enzyme catalyses the NADPH-dependent reduction in the redox protein Trx [237], thereby impairing cellular protective mechanisms against oxidants [235]. However, Trx depletion affects gene expression by modulating the DNA-binding activity of specific transcription factors [238,239,240]. A decrease in cellular GSH content sensitises cultured rat myoblast (L6) cells to *i*As^(III)^ and *i*As^(V)^ compounds, contributing to cell transformation [241].

*i*As can induce mitochondrial biogenesis through the mitochondrial transcription factor A (mtTFA) in keratinocytes, the primary cell type of the epidermis. However, *i*As also induces mitochondrial dysfunction and apoptosis in the hearts of ducks by inhibiting Nrf2 activation, downregulating Nrf2, HO-1, and SOD-1 *m*RNA and protein, and upregulating the expression of Keap1 *m*RNA and protein [242]. Exposure to *i*As also increases mitochondrial oxidative damage (OD) with mitochondrial DNA (*mt*DNA) mutation in keratinocytes and tumour tissues of patients with *i*As-induced skin cancers. Thus, carcinogenesis progression is related to mitochondrial OD, making mitochondria a therapeutic target for treating cancers involving *i*As^(III)^ [243].

Furthermore, *i*As suppresses the longevity gene *SIRT1*, which modulates fibrous actin in the liver and MIHA cells, thereby impeding the formation of membrane structures, such as tunnelling nanotubes, and the mitochondrial transfer between cells [244]. Specific demethylase fat mass and obesity-associated protein (FTO) can catalyse high levels of N6-methyladenosine, regulating OD induced by *i*As via reader protein IGF2BP3 (insulin-like growth factor 2 mRNA binding protein 3). This process represents a new molecular mechanism underlying FTO-mediated OD, implicating enhanced SUMOylation of the FTO protein and thereby reducing its expression, which serves as a potential approach for treating OD [245]. Note that SUMOylation is a post-translational modification involving a family of small proteins (SUMO, Small Ubiquitin-like MOdifier) covalently attached to and detached from other proteins, modifying their function and playing a role in mediating protein quality control in cardiomyocytes by stimulating degradation through the ubiquitin proteasome system [246].

The action of *i*As on NAD-linked substrates (support of mitochondrial respiration, decrease in adenosine triphosphate (ATP) synthesis, and increase phosphorylation of other intracellular biochemical species) also generates ROS (i.e., one-electron reduction of O_2_ to form free radical superoxide, O_2_^•^), inducing OS, and altering cellular gene regulation [113,247,248,249]. ROS production can occur by activating the enzyme NADPH oxidase (NOX), which transfers electrons from NADPH to oxygen, generating reactive O_2_^•^, and hydrogen peroxide (H_2_O_2_). However, the enzyme NOX generates more ROS in the presence of *i*As, and the subunit p22phax, which is responsible for electron transfer, was upregulated by *i*As [26]. The *i*As^(III)^ ion in cells induces the production of H_2_O_2_ as a result of the previous formation of ROS, such as ^•^OH and ^•^OOH [249], probably via Fenton- and Haber–Weiss-type reactions (Reactions (3)–(7)),

*i*As^(III)^ + 2 H_2_O_2_ → *i*As^(V)^ + 2 ^•^OH + 2 OH^-^ (3)

*i*As^(V)^ + 2 H_2_O_2_ → *i*As^(III)^ + 2 ^•^OOH + 2 H^+^ (4)

*i*As^(V)^ + 2 O_2_^•^ → *i*As^(III)^ + 2 O_2_ (5)

(CH_3_)_2_AsH + O_2_ → (CH_3_)_2_As^•^ + O_2_^•^ + H^+^ (6)

(CH_3_)_2_As^•^ + O_2_ → (CH_3_)_2_AsOO^•^ (7)

The enzyme NOX contributes to the As^(III)^-dependent oxidation of PARP-1 {poli-(ADP-ribose)-polymerase-1}, thus inhibiting PARP-1 activity and enhancing UVR-induced DNA damage in keratinocytes. In human skin, the enzyme NOX2 is expressed at greater levels than the enzyme NOX1 [250,251,252], demonstrating that the NOX2 isoform is the major contributor to the As^(III)^-induced ROS and DNA damage retention in keratinocytes. NOX2 [253] and also metals such as zinc [254] show a role in mediating the effects of As^(III)^ on UVR-induced DNA damage repair. The As^(III)^ inhibition of UVR-induced PARP activity is not evident in the epidermis of NOX2^−/−^ mice compared to controls, and retention of UVR-induced DNA damage by As^(III)^ decreases in NOX2^−/−^ mice [253].

On the other hand, DMA^(III)^ also forms ROS by reaction with O_2_, generating the dimethyl arsenic radical {(CH_3_)_2_As^•^} and the dimethylarsenic peroxyl radical {(CH_3_)_2_AsOO^•^} [255]. Further, both DMA^(III)^ and DMA^(V)^ at 10 mmol/L concentration in vitro can release iron from horse spleen and human liver ferritin, at high rates (29.8 nmol/L Fe^2+^/min) as compared to the other arsenic species in the presence of ascorbic acid, thus promoting ROS through the Fenton reaction [164]. ROS stresses the endoplasmic reticulum, increasing the amount of unfolded protein response signals, which leads to inflammation, cell proliferation, and ultimately, cell death through cytoskeleton rearrangement and the disruption of contractile proteins [26]. Nonetheless, ROS also exhibits positive effects as a regulatory agent [256]. However, accurate analytical methods are available for determining ROS in cells and in vivo, thereby enhancing our understanding of their biological functions [257].

RNS, which is responsible for damaging DNA, forms after ROS damage to the mitochondria [26]; however, other unstable, reactive species are also typically present. Thus, the endothelial NOS combines with L-arginine to form NO, but MMA^(III)^ at concentrations of 0–15 μmol/L inactivates this enzyme in a concentration-dependent manner [26] by reducing the NO generation and its bioavailability. Similarly, As^(III)^ also acts as a competitive inhibitor, interfering with the binding of L-arginine, a substrate, to the active site of NOS, thereby reducing NO production. NO participates in cell regulation, including cellular metabolism, growth, division, and even death [26]. Furthermore, the superoxide O_2_^•^ can react with NO radicals [26], releasing the much more cytotoxic peroxynitrite (ONO_2_^−^), an unstable structural isomer of nitrate.

*i*As^(III)^ affects voltage-gated potassium channels [258], which can cause life-threatening heart rhythm problems due to low cellular potassium levels. Consequently, disrupting cellular electrolytic function results in neurological disturbances and several cardiovascular episodes, such as a wider QT interval on electrocardiogram (elapsed time taken by the heart muscle to contract and recover), neutropenia (abnormally low levels of neutrophils), high blood pressure, central nervous system dysfunction, anaemia, and death [259]. *i*As compounds block both IKr and IKs channels in vitro with an IC_50_ for tail current block of 0.14 ± 0.01 µmol/L for IKr and 1.13 ± 0.06 µmol/L for IKs but activate IK-ATP channels and disrupt ATP production in HERG- or KCNQ1+KCNE1-transfected CHO cells [260]. Alternatively, at the citric acid cycle level, the binding between MMA^(III)^ and the thiol group of the lipoic acid co-factor allosterically inhibits the activity of the essential PDH complex [183], which catalyses pyruvate oxidation to acetyl-CoA with the transformation of NAD^+^ to NADH, uncoupling oxidative phosphorylation by competing with PO_4_^3−^. Consequently, it inhibits the energy-linked reduction in NAD^+^, mitochondrial respiration, and ATP production in the electron transport chain, as well as gluconeogenesis intermediates [183]. The cell’s energy system is disrupted in this situation, leading to a cellular apoptosis episode.

*i*As is a significant environmental risk factor for neurodegenerative diseases, triggering neuronal cell death through the following signalling pathways: (i) the JNK/ERK activation-mediated mitochondria-dependent apoptosis pathway, where mitochondrial dysfunction, PARP cleavage, and caspase cascade activation are essential for apoptosis, and (ii) the GRP 78 and CHOP activation resulting in neuronal cell apoptosis [261]. As^(III)^-induced upregulation of AS3MT, which is overexpressed in human lung adenocarcinoma (A549) and human bronchial epithelial (16HBE) cells, inhibits the p53 signalling pathway by the deactivation of p38 MAPK and binding to c-Fos, thereby promoting cell proliferation and inhibiting cellular apoptosis. This way, AS3MT might be a notable proto-oncogene mediating *i*As-induced tumorigenesis in lung cancer [262]. Alternatively, the *i*As-induced transformation (As-T) cells show upregulation of EGFR (epidermal growth factor receptor) and downregulation of miR-218-5p. MiR-218-5p can act as a tumour suppressor, inhibiting cell proliferation, migration, colony formation, tube formation, tumour growth, and angiogenesis by directly targeting EGFR via its 30-untranslated region (UTR). This way, the 218-5p/EGFR signalling pathway represents a potential therapeutic target for treating lung cancer induced by chronic exposure to *i*As [263]. Furthermore, *i*As, which is linked to N6-methyladenosine (m6A), a significant and dynamic epigenetic RNA modification, contributes to the development of *i*As^(III)^-induced skin lesions. Arsenite induces METTL3 (methyltransferase-like 3) upregulation, represses suppressors of cytokine signalling 3 (SOCS3) expression in an m6A-YTH m6A RNA binding protein 2 (YTHDF2)-dependent manner, and leads to the aberrant activation of the Janus kinase (JAK)2/signal transducer and activator of transcription 3 (STAT3) signalling pathway. The activated transcription factor STAT3 binds to the promoters of Krt1 and Krt10, thereby facilitating their transcription, and ultimately leading to As^(III)^-induced skin lesions [264].

On the other hand, *i*As^(V)^ species bind covalently to the thiol groups of GSH and Cys residues, forming thioesters [124]. They also react with glucose and gluconate to form glucose-6-arsenate and 6-arsenogluconate, which act as analogues for glucose-6-phosphate and 6-phosphogluconate, respectively [183]. Unlike the importance of PO_4_^3−^ in glycolysis, *i*As^(V)^ attacks the enzyme-bound thioester in the D-glyceraldehyde-3-phosphate dehydrogenase reaction, forming anhydride 1-arsenate-3-phospho-D-glycerate, which hydrolyses spontaneously due to the long As-O’s bond length (≈1.77 Å) compared to P-O (≈1.54 Å) [183]. As a result, it prevents the subsequent transfer of PO_4_^3−^ to adenosine diphosphate (ADP) and impedes ATP formation in glycolysis [183], thereby lowering cellular energy. These metabolic interferences can lead to death due to a multi-system organ failure, probably necrotic cell death, but not apoptosis. Submitochondrial particles synthesise adenosine-5′-diphosphate-arsenate from ADP and *i*As^(V)^ in the presence of succinate, impairing cell respiration and subsequently diminishing ATP formation [265]. Chronic exposure (18–20 weeks) of CAsE cells to low levels (500 nmol/L) of *i*As^(III)^ species also induces the production of proteins, such as metallothionein (MT-I/II) in a rat liver epithelial cell line (TRL 1215) [266,267], which in turn gives protection against proper *i*As^(III)^ toxicity, mainly because MT blocks the possible participation of intracellular heme group and porphyrins in the formation of ROS. Thus, levels of GSH, ferritin, porphyrins, and the 32 kDa stress protein (heme oxygenase, HO) increase after treatment of human cells with *i*As^(III)^ [208,268,269,270]. Other stress proteins, such as those in the 27, 60, and 90 kDa families, are also induced by *i*As^(III)^, indicating broad proteotoxic stress (accumulation of misfolded proteins) [271].

##### Chronic Effects (Chronic Exposure) of Arsenic Species to Humans

The chronic effects result from a slow but permanent accumulation of low doses of toxicants. Documentation of the chronic effects of *i*As in humans is extensive, with the most affected organs usually involved in absorption, accumulation, and excretion (the gastrointestinal tract, circulatory system, heart, liver, kidney, and skin) [272]. Strong epidemiological relationships exist between long-term ingestion of *i*As and bladder, lung, kidney, and liver cancers, as well as other health effects [273,274,275]. Thus, prolonged exposure to low-level *i*As elicits a pro-inflammatory profile, partly driven by the blood monocyte marker CD14, which can lead to the prolonged persistence of pulmonary and systemic inflammation, promoting oxidative DNA damage in rural women [276]. Chronic exposure to *i*As relates to vitamin A deficiency, heart disease (hypertension-related cardiovascular disease) [277], stroke (cerebrovascular diseases) [278], chronic lower respiratory diseases [279], diabetes [280,281], and night blindness (nyctalopia) [282]. The most frequent signs of chronic *i*As toxicity include dermal lesions (hyperpigmentation, hyperkeratosis, desquamation or skin peeling, and loss of hair), peripheral neuropathy (damage of the peripheral nerves), Blackfoot disease (BFD, a peripheral vascular disorder), and different cancers [210,283]. However, each cancer type shows a different *i*As speciation profile and, in general, a different metallome [284]. All skin cancers, including in situ cell carcinoma (Bowen’s disease), invasive cell carcinoma, and multiple basal cell carcinomas, are associated with chronic *i*As exposure [285,286,287]. These signs have been observed chiefly among people working in arsenic ore smelters, pesticides, and drinking water contaminated with *i*As [239], representing a public health issue worldwide [173]. Individuals who chronically drank water contaminated with *i*As showed increased cytokine expression and cell proliferation in skin biopsies [288]. A correlation exists between chronic *i*As exposure through drinking water and Type 2 diabetes mellitus [87], although no scientific evidence exists regarding cause-and-effect relationships. Nonetheless, related findings have shown a direct correlation between elevated serum lipid peroxides (LPO) and blood levels of *i*As and its methylated metabolites in individuals exposed to *i*As, but an inverse correlation with nonprotein sulfhydryl levels in the general population [289]. Nevertheless, evidence linking arsenic exposure to chronic kidney disease is still scarce and limited to some populations [290].

Notwithstanding these factors, several other factors, such as inadequate nutrition and genetics, can also enhance susceptibility to different *i*As-induced diseases [282,291,292]. Thus, Euro-Americans show basal cell carcinoma, but in Asians, Bowen’s disease. On the other hand, individuals from India show a range of health effects, including peripheral vascular disease (BFD), non-cirrhotic portal fibrosis, obstructive lung diseases, and polyneuropathy (peripheral neuropathy), whilst in Taiwan (China), changes in skin pigmentation and hyperkeratosis are widespread [293]. The vascular disease appeared among German vintners, inhabitants of Antofagasta (Chile), and Taiwanese people [294]. Other effects are hematopoietic depression, anhydremia (abnormal reduction in water in the blood), liver damage characterised by jaundice (icterus), portal or nutritional cirrhosis (Laënnec’s cirrhosis) and ascites, sensory disturbance and peripheral neuritis, anorexia and weight loss. Females are more susceptible to developing *i*As-induced skin affection, probably due to a more efficient methylation process in females’ *i*As metabolism [295,296]. Thus, Thai females are more affected by skin manifestations, including skin cancer, which is likely induced by the combined effects of skin melanin and ultraviolet (UV) radiation [297].

Some epigenetic effects change gene expression without altering the genetic code by altering DNA methylation [298], which can result in opposite methylation patterns (hypo- and hyper-methylation). The identification of signature epigenetic patterns associated with telomere lengthening, mitochondrial functions, and DNA damage repair in arsenic-exposed children revealed altered DNA methylation profiles, including promoter hypermethylation of MLH1 and MSH2, indicating inefficiency in DNA damage repair. Additionally, hypomethylation was observed in the mitochondrial D-loop and the TFAM promoter region, accompanied by an increase in the number of mitochondrial DNA copy number. Further, a significant increase in telomere length and region-specific subtelomeric hypermethylation was found. Analysis of SAM and 8-oxo-2′-deoxyguanosine (8-oxo-2dG) levels revealed significant depletion of SAM and elevated oxidative DNA damage in *i*As toxicity [299].

Choline and other methyl-group donors, such as methionine, betaine, and the B group vitamins, influence DNA methylation primarily by affecting the concentrations of SAH and SAM. Thus, hypomethylation may occur due to a lack of SAM, resulting in aberrant gene activation [98,300,301]. In mice, making hepatic methyl donors deficient through a choline-deficient diet, arsenite-induced genotoxicity shifts from the liver and bladder (in normal mice) to the skin (in methyl-deficient mice) [302]. The long-term effects of *i*As suggest that genome-wide DNA hypomethylation contributes to genetic instability [303]. Chronic low-dose As^(III)^ exposure can lead to global hypomethylation, which is associated with prolonged exposure periods, accompanied by the concomitant induction of the DNA methyltransferase genes *DNMT1* and *DNMT3B*, and a slight downregulation of DNMT3A. Thus, pronounced time- and concentration-dependent effects of *i*As are observed when genes are involved in the DNA damage response and repair, inflammation, the OS response, and metal homeostasis are affected [304]. Hypomethylation also represents a critical process in the transformation induced by *i*As^(III)^-induced transformation of Syrian hamster embryo cells [305].

The interaction of As^(III)^ with methyltransferases, particularly in relation to DNA methylation, typically leads to the inactivation of tumour suppressor genes through hypermethylation [306]. Many proteins encoded by genes with differentially methylated cytosine-guanine (CpG) islands play critical roles in arsenic-associated diseases, including heart disease, diabetes, and cancer. Chronic exposure to *i*As^(III)^ leads to a large interactome of hypermethylated genes, including *p53* and *p16*, which are involved in cancer-associated pathways [307,308]. Nonetheless, conclusive results on the effect of *i*As^(III)^ on DNA methylation of the *p53* gene promoter in human lung A549 carcinoma cells are still elusive. In other words, progressively increased methylation at CpG islands—DNA sequence clusters rich in CpG dinucleotides within the *p53* promoter—would be expected to block transcription of the *p53* gene [309,310]. However, the protein produced by the tumour-suppressor *p53* gene functions as a transcriptional regulator, playing a crucial role in activating the expression of numerous other genes involved in cell death, cell cycle arrest, senescence, apoptosis, DNA repair, and other processes [311,312]. Cells treated with *i*As^(III)^ reported altered expression of the *p53* promoter and changes in the cell cycle distribution, while cells transfected with a mutant *p53* gene showed increased sensitivity to *i*As^(III)^ cytotoxicity [206,313]. An arsenic-methylated tumour suppressorome comprised seventeen known or putative tumour suppressors, silenced in arsenic-induced human cancers (bladder, kidney, lung, liver, and prostate), cardiovascular disease, and diabetes mellitus [308,314,315].

Different altered growth factors lead to cell proliferation and carcinogenesis [48]. Thus, cell proliferation induced by mitogenic stimuli or by regeneration after cytotoxicity can enhance carcinogenesis [316]. No definitive model exists for the Brambila chronic *i*As poisoning mechanism, although chronic low-dose *i*As poisoning may increase tolerance to its acute toxicity [207,283], mediated through the drug pump MRP1-mediated efflux [317]. In GLC4 and GLC4/ADR cells, overexpression of MRP1, a human membrane protein, reduced the efficiency of arsenic-derived anticancer drugs. Furthermore, as the MRP1-catalysed export of GSH from cells increases in response to As^(III)^ and its efflux depends on cell type (resistant or sensitive), it has been postulated that MRP1 can act as a carrier of the As(SG)_3_ complex [318,319]. However, its significance in the in vivo metabolism and transport of As^(III)^ still needs to be demonstrated [320]. The toxicity and carcinogenicity of *i*As might be tissue-specific, whilst co-carcinogenicity occurs in diverse models. *i*As^(III)^, and perhaps some of its metabolites, can act as co-carcinogens by activating signal transduction pathways, enhancing cell proliferation, reducing antiproliferative signalling, and overriding checkpoints controlling cell division after a genotoxic insult [113]. The current consensus on the mode of carcinogenesis is that it acts primarily as a tumour promoter. However, Andean populations (most highly exposed to UV radiation) do not develop skin cancer with chronic *i*As exposure [167], probably due to differences in the levels of *i*As in the drinking water, although concentrations of 1.25 mg/L *i*As^(III)^ are able to enhance the tumorigenicity in mouse skin of solar UV irradiation, the most effective carcinogenic partner of arsenic [321]. Some of the factors governing the differences existing in the generation of skin cancer by As can include different UV-induced genotoxic photoproducts in DNA to those usual (cyclobutane dimers and 6–4 photoproducts at dipyrimidine sites), oxidative lesions, differences in the epigenetic influence, differences in the methylation process, DNA repair, DNA methylation, enhanced proliferation, and induction of genomic instability via effects on telomerase and the enzyme poly(ADP-ribosyl) polymerase [322]. Several mitogens (mitotic inducers), including growth factors, mitogenic lipids, inflammatory cytokines, and hormones, are modified by integrin-mediated adhesion (a critical regulator of cell migration) and thereby regulate the proliferation of mammalian cells [323].

As^(III)^ impairs the immune system [108,324], thereby increasing susceptibility to viral and bacterial infections. Some viral infections, like the human papillomavirus (HPV), represent an additional risk factor in developing non-melanoma skin cancer among populations chronically exposed to *i*As [325] and with squamous cell carcinoma and keratosis [326]. The unfolded protein response (UPR) increases the activity of several receptors responsible for restoring homeostasis [108]. The two receptors, inositol-requiring enzyme-1 (IRE1) and protein kinase RNA-like endoplasmic reticulum kinase (PERK), regulate the translation rates. On the other hand, chaperones correct the unfolded proteins induced by the activating transcription factor 6 (ATF6). If the number of erroneous proteins increases, another mechanism activates apoptosis, whose activity is enhanced by *i*As [108]. Apoptosis may be an essential mechanism for *i*As-induced immunosuppression [327]. However, malfunctions in apoptosis can lead to various diseases, including cancer [328]. Substantially low *i*As^(III)^ concentrations trigger apoptosis in B cell line TA3, which could mediate immunosuppressive effects [324,329]. *i*As causes anaemia [286], whose severity indicates the extent of disruption to standard regulatory mechanisms exerted by macrophages and T-cells [330]. In children, *i*As disrupts the ratio of CD4+ helper to CD8+ cytotoxic T cells, which is responsible for immunodepression [265], and increases the number of inflammatory molecules secreted through macrophages. An excessive number of granulocytes and monocytes can lead to chronic inflammation, which may contribute to the development of cancer [265].

##### Genotoxicity and Oxidative DNA Damage

The replacement of phosphorus (PO_4_^3−^) with arsenic (AsO_4_^3−^) in DNA or RNA is not straightforward and requires additional experimentation [331,332,333,334]. *i*As is a weak mutagen because low *i*As^(III)^ concentrations do not react directly with DNA, even for bacteria [122]. Genome stability is notably altered through DNA replication, particularly when unrepaired or poorly repaired damage occurs. DNA repair enzymes are insensitive to inhibition by *i*As^(III)^, and consequently, *i*As^(III)^ can likely interfere with the regulation of DNA repair rather than with the specific repair enzymes [322]. Significant consumption of cooked rice with *i*As concentrations exceeding 200 µg/kg generates genotoxic effects in humans, as measured by MN in urothelial cells [335]. Indirect genotoxicity in diverse in vitro and in vivo systems involves cytotoxic exposure, which is not the basis for cancer development. Evidence for genotoxicity in humans involves the detection of chromosomal aberrations, sister chromatid exchanges and micronucleus formation in lymphocytes, buccal mucosal cells, and exfoliated urothelial cells in the urine. However, a non-linear dose–response relationship exists for the effects of *i*As^(III)^, although with a threshold for biological responses, including cancer, in diverse in vitro and in vivo animal models and human epidemiological studies [336]. Overall, *i*As^(III)^ genotoxicity involves gene amplification and chromosomal damage, enhancing the mutagenicity of other carcinogens [337], such as UV light in humans and other mammalian cells [338], probably by inhibiting DNA methylation and DNA repair [339], which is one of the main mechanisms of genotoxicity involving *i*As^(III)^. Thus, *i*As^(III)^ species interfere with the base and nucleotide excision repair [340], inhibiting the repair of endogenously produced lesions, such as spontaneous mutagenesis caused by increasing endogenous reactive oxidant levels, ROS and NO, in plasma [341,342,343], directly associated with *i*As levels in whole blood [344]. The *i*As genotoxicity generates ROS [345,346,347], which can cause aberrant gene expression at low concentrations but, at higher concentrations, can damage lipids, proteins, and DNA (adducts, strand breaks, cross-links, and chromosomal aberrations) [348,349,350,351]. ROS also induces DNA base damage through nucleotide excision repair (NER) and base excision repair (BER). NER repairs bulky distortions in the DNA double helix while BER mainly focuses on single-strand breaks. Arsenic species (*i*As^(III)^, MMA^(III)^, and DMA^(III)^) at concentrations up to 500 μmol/L, incubated in vitro for 24 h with human A549 epithelial lung adenocarcinoma cells, induced NER and BER inhibition, much more strongly with mixed arsenic species, although DNA polymerase beta showed an essential role in reducing arsenite-induced DNA damage [352,353,354,355,356,357].

OD occurs by modifying DNA nucleobases, particularly 8-oxoguanine (8-hydroxyguanine, 8-oxo-Gua, OH8Gua, 8-OHdG), which leads to G-C and T-A mutations [358]. *i*As can cause malignant transformation in cells that do not methylate it, indicating that arsenic methylation is not necessary for the development of a malignant phenotype. However, methylated arsenic species, such as MMA^(III)^, are directly involved in the generation of DNA OD [205]. Biomethylation of arsenic is essential for generating oxidative DNA damage, thereby increasing carcinogenicity. Ogg1 genetic background and arsenic-induced 8-OH-dG proved relevant for *i*As-mediated carcinogenic effects [359]. Nonetheless, MT has high Cys content and can protect against *i*As-induced OD, possibly by sequestering ROS or by directly responding to *i*As. This effect is minor in cells that produce MT, showing minimal sensitivity to *i*As-induced OD [360]. Compounds, such as polydatin (3,5,4′-Trihydroxystilbene-3-O-β-D-glucopyranoside) or piceid (a stilbenoid glucoside precursor of resveratrol), have a dose-dependent protective effect on *i*As-induced LPO DNA damage, inflammation, and apoptosis in rats [361], enhancing the activity of the antioxidant defence system.

*i*As^(III)^, but not *i*As^(V)^, species induce DNA double-strand breaks in both lung fibroblasts and epithelial cells but only induce chromosomal aberrations, occasionally including chromosome breaks too [362], endoreduplication (replication of the nuclear genome without mitosis), aneuploidy (abnormal number of chromosomes), mitotic abnormalities, deletion mutations, MN formation [363,364], and cross-linking of DNA with proteins [113,365,366], particularly in human fibroblasts. Nonetheless, both *i*As^(III)^ and *i*As^(V)^ can cause sister chromatid exchanges [367]. *i*As cause concentration-dependent but not time-dependent chromosome damage in lung fibroblasts, indicating cytotoxicity and genotoxicity in human lung primary cells. However, lung fibroblasts are more sensitive to the cytotoxicity of *i*As than epithelial cells [368]. Protein cross-links might be the primary DNA lesions induced by *i*As^(III)^ [268,347,351,369,370]. Unlike spindle toxins, which disrupt cell division by altering the protein threads and connecting the centromere regions of chromosomes, *i*As^(III)^ species do not inhibit spindle fibre formation; instead, they upset the spindle apparatus (mitotic spindle), possibly by accelerating microtubule polymerisation [371]. Compounds, such as TiO_2_ nanoparticles (NPs), enhance the genotoxicity of As^(III)^ in human–hamster hybrid mammalian cells via physicochemical interactions mediated by mitochondria-dependent ROS [372].

*i*As causes epigenetic effects involving DNA methylation, histone tail modifications, and microRNA activity (noncoding RNA that modulates mRNA translation), which, together with its capacity to induce mutations, could be the leading cause of *i*As-induced carcinogenesis [373].

Large doses of DMA^(V)^ (up to 400 mg/L DMA^(V)^ in drinking water) cause DNA damage in male F344/DuCrj rats, acting as a tumour promoter in mouse lungs, previously initiated with 4-nitroquinoline 1-oxide [121]. DMA^(V)^ in rats produce high levels of 8-hydroxy-2′-desoxyguanosine (8-OHdG, a possible biomarker of ROS DNA damage) in the urine [107]. Further, DMA^(V)^ at 5–100 μmol/L concentrations causes DNA single-strand breaks in human epithelial type II cells [351,374], also inducing DNA-protein cross-links and alkali-labile sites (a weakened polymer structure at the site of the attack on DNA) in cultured human alveolar L-132 cells [375,376]. MMA^(III)^ and DMA^(III)^ are directly genotoxic by effectuating scissions in supercoiled ΦX174 DNA [178].

### 2.4. Beneficial Effects of Inorganic Arsenic

*i*As can cause toxicity but also play protective roles in various ways [377]. *i*As can generate a potent poison, and some monomethylated arsenicals, such as MMA^(V)^, acted as essential precursors in the preparation of earlier pesticides, insecticides (Paris green), herbicides, and fungicides (Neoasozin), even though they show diverse illnesses in humans [378].

Nonetheless, the use of *i*As for over 200 years has also been reported to have direct beneficial effects on humans, particularly in traditional Chinese medicine, prior to the development of penicillin [379,380]. Thus, Fowler’s solution {1% potassium meta-arsenite (KAsO_2_)} and Donovan’s solution {arsenic triiodide and mercuric iodide (AsI_3_ and HgI_2_) mixed with sodium bicarbonate (NaHCO_3_)} have historically been reported (in the western world) as remedies for stomach ailments, several digestive problems, rheumatism, psoriasis, and syphilis [381].

The first synthetic chemotherapeutic agent applied as an antibiotic derives from the organoarsenic compound arsphenamine (Salvarsan, compound 606, Solarson, 2-amine-4-(3-amine-4-hydroxyphenyl)arsanilidenearsanilphenol clorhydrate) (Table 2), known as the “silver bullet” [382]. Later, sulfa drugs and other antibiotics replaced salvarsan as therapeutic agents. On the other hand, melarsoprol (Mel B; 2-(p-(4,6-diamino-S-triacin-2-ilamino)phenyl)-1,3,2-dithiarsolane-4-methanol) was an available medicinal compound for the treatment of trypanosomiasis (African sleeping sickness) related to the human central nervous system [382,383]. Mel B results from a condensation reaction between melarsen oxide (2-N-(4-arsorosophenyl)-1,3,5-triazine-2,4,6-triamine) and Dimercaprol or British anti-Lewisite (BAL, (R,S)-2,3-Dimercaptopropanol).

On the other hand, *i*As^(III)^ inhibits the in vitro proliferation of myeloma cells by inducing cell cycle arrest and triggering cell death [384], suggesting a potential clinically sound treatment for patients with multiple myeloma [385] or leukaemia [386]. *i*As^(III)^ induces the mitochondrial pathway of apoptosis in human leukaemia (HL-60) cells. However, this process is modulated by OS, DNA damage, and changes in the mitochondrial membrane potential, resulting in the translocation and upregulation of apoptotic proteins and programmed cell death [387]. *i*As^(III)^ is a chemotherapeutic drug for treating acute promyelocytic leukaemia (APL). Some patients with APL are often refractory to retreatment with tretinoin or all-*trans* retinoic acid (ATRA) and anthracycline chemotherapy [388], which is the first-line treatment; however, they respond successfully to the treatment with *i*As^(III)^ [381,389,390]. *i*As^(III)^ induces partial cytodifferentiation [389,391] and also triggers apoptosis of leukaemia cells, resulting in high remission rates in patients with relapsed APL [392] and chronic lymphocytic leukaemia [393]. Different diseases, including hyperleukocytosis, APL differentiation syndrome, electrocardiographic abnormalities, and hyperglycemia, were manageable [394], resulting in substantial clinical benefits. No increase in the incidence of secondary malignancies was observed among APL patients who received *i*As^(III)^ up to 5 years of follow-up [395]. Furthermore, moderate to high doses of *i*As^(III)^ and Mel B can control some leukaemia types (i.e., leukocytosis in patients with chronic myelocytic leukaemia) [383,384] or, in cooperation with lonidamine, induce apoptosis in HL-60 and other human leukaemia cell lines, with low toxicity in non-tumour peripheral blood lymphocytes [396], but sudden deaths among some patients also occur [397,398,399]. *i*As^(III)^ can act as a therapeutic agent, but it generates other side effects [400], as mentioned above.

Nonetheless, substantial efforts to expand its clinical utility to solid tumours are challenging [401] because *i*As^(III)^ still present many drawbacks in clinical use, mainly including rapid renal clearance and short half-life, severe adverse effects, and high toxicity to normal cells (folate receptor (FR)–positive human nasopharyngeal (KB) and cervix (HeLa) cells). However, nanomedicine can offer potential solutions to these limitations, treating them to achieve biocompatibility, targeting capability, and desirable effectiveness [402]. In this way, *i*As^(III)^ and ascorbic acid synergistically inhibit the viability of human colorectal cells, activate caspase-3 to trigger apoptosis, upregulate caspase-1 expression, and promote the formation of inflammasomes to induce pyroptosis. Furthermore, the combined treatment with *i*As^(III)^ and ascorbic acid stimulates the overproduction of ROS, a subcellular mechanism underlying apoptosis and pyroptosis [403]. Supplementation to rodent models with vitamins folate (B9) and cobalamine (B12) acts against arsenic-related tissue/DNA damage. Furthermore, they protect RBCs and maintain the expected levels of haemoglobin and other compounds, such as urate, mercaptans, and diverse enzymes (catalase, GSH peroxidase, peroxiredoxins, SOD, lactoperoxidase, and xanthine oxidase) [404]. As_2_O_3_-NP encapsulated in folate-targeted liposomes reduce toxicity systematically, providing a platform for targeted delivery of this agent. These arsenic “nanobins” release drugs when the pH decreases to endosomal or lysosomal levels. *i*As^(III)^ is also a devitalizing agent in tooth cavities [381,405]. As_2_O_3_-NPs promote more outstanding LDH release and induce pyroptosis. However, As_2_O_3_-NPs decrease the expression of the proteins, Dnmt3a (DNA(cytosine-S)-methyltransferase-3α), Dnmt3b (DNA(cytosine-S)-methyltransferase-3β), and Dnmt1 (DNA(cytosine-S)-methyltransferase-1), but inhibit tumour growth more strongly than As_2_O_3_, probably due to the downregulation of PCNA (proliferating cell nuclear antigen) and DNMT-related proteins and the upregulation of GSDME-N (Gasdermin E-N) [406].

Low doses of *i*As can also benefit animals, acting as a growth factor, particularly in chicken nutrition [173]. The primary uses of some derivatives of phenylarsonic acid (C_6_H_5_AsO(OH)_2_), including 4-hydroxy-3-nitrobenzene arsonic acid (roxarsone), 4-ureidophenyl arsonic acid (carbarsone), and *p*-arsanilic acid, were as feed additives for livestock [43]. Other applications of arsenic derivatives include copper arsenates as a preservative for treating wood and their use externally in mineral hot springs. Arsenic reportedly protects against Se toxicity, enhancing the biliary excretion of Se [406]. Thus, at lethal dose Se and As^(III)^ interact to form an equimolar arsenic−selenium compound in the liver, which is then excreted in the bile of rabbits, identified spectroscopically as [AsSe(SG)_2_]^−^ [161,195]. However, it is worth noting that the beneficial and harmful effects depend on the metabolic pathways involved in the excretion or accumulation of arsenic species and possibly also on potential interactions with other compounds.

## 3. Diagnosis and Possible Treatment

### 3.1. Diagnosis

To monitor high environmental or occupational exposures and to confirm poisoning for forensic purposes, the primary diagnosis of intoxication by *i*As mainly relies on measuring its levels in body fluids (blood and urine), hair, and fingernails, as well as on the cutaneous manifestations (melanosis, keratosis, and cutaneous cancers). Urinary porphyrin levels and blood MT show promising results as potential biomarkers of *i*As exposure [407,408,409]. Thus, increased urinary B2MG (β2-microglobulin) concentrations show a significant relationship with urinary *i*As^(V)^ concentrations in Taiwanese adults (aged 20–29 years) [410]. Elevated *t*As levels in the urine of Chinese adults were associated with increased annual change rates in the levels of 8-isoPGF2α (8-iso prostaglandin F2α) lipid peroxidation, 8-OHdG (8-hydroxy-2′-deoxyguanosine) DNA OD, and PCO (protein carbonyls) protein OD in dose-dependent manners [411]. It is usual to distinguish between the following three possible biomarker types: (i) exposure, (ii) effect, and (iii) susceptibility. Thus, porphyrins and *t*As in the urine, blood, hair, and nails represent typical biomarkers of exposure. The effects’ biomarkers include clastogenicity in peripheral lymphocytes, MN in oral mucosa and bladder cells, and induction of heme oxygenase. Finally, the biomarkers of susceptibility typically account for variability in *i*As metabolism [96,412,413].

Genomics [126] and proteomics [127] have also facilitated the identification of potential biomarkers for *i*As toxicity. Reconstructed genes from a reference transcriptome of the aquatic oligochaete, *Tubifex tubifex* (Annelida, Clitellata), relate to cell stress response (Hsc70, Hsp10, Hsp60, and Hsp83), energy metabolism (COX1), OS (Cat, GSR, and MnSOD), and the homeostasis of organisms (CaM, RpS13, and UBE2) can be used for risk assessment in freshwater ecosystems as early biomarkers of arsenic toxicity [414]. Changes in the urinary proteome have been shown to help identify potential *i*As-associated pathologies and elucidating the mechanisms involved in chronic exposure to *i*As [415,416,417]. Alternatively, monitoring malondialdehyde levels, enhanced by *i*As, can serve as a valuable marker for OS, although other toxicants can also contribute in the same direction.

Potential biomarkers for *i*As intoxication in populations exposed to *i*As in drinking water include the following:

1. The arsenic level in the blood measures the dose ingested but does not provide evidence of chronic intoxication.
2. The arsenic level in urine is a reliable marker of internal dose, and correlates well with chronic effects from *i*As in drinking water, although it requires detailed speciation analysis.
3. Arsenic level in hair and nails reflects long-term exposure.
4. The As content in urine correlates with urinary and blood porphyrin levels.
5. Genotoxic effects, including increased DNA damage, sister chromatid exchange, micronuclei, or chromosomal aberrations, support the DNA OD of peripheral blood polymorphonuclear leukocytes and
6. MN assays are the techniques of choice for assessing the adverse health effects of low levels of *i*As as essential biomarkers.

However, urinary and toenail analysis are usually the most practical sampling choices, complemented by MN assays [418,419]. The most reliable test for *i*As exposure is urine-based, but it must be performed within 24–48 h to detect acute exposure accurately. According to the biomarker 8-OHdG, *i*As in urine is associated with an increased prevalence of type 2 diabetes mellitus, a mechanism partly involving DNA OD but lacking a causal relationship [420]. The same holds for the lack of relationships between the blood concentration and the effect of oxidation states on ROS production, DNA fragmentation, and apoptotic cell death.

Tests on hair and fingernails are adequate for measuring exposure to high levels of arsenic during long periods (3–6 months) [421]. However, they cannot predict whether arsenic levels in the body have a health impact. In chronic exposures, *i*As remains in the body for very long periods. Arsenic levels in hair serve as a reliable bioindicator of *i*As exposure, as they accumulate trace elements from the blood. Incorporated elements in hair are maintained during hair growth, allowing a temporal exposure estimation. Hair sampling facilitates the determination of arsenic levels, and the treatment of APL with arsenic oxide [422]. For this purpose, non-invasive microanalytical techniques offer attractive advantages, including X-ray fluorescence (XRF) using electromagnetic radiation (synchrotron radiation), electron or particle beams as excitation probes, such as particle-induced X-ray emission (PIXE), FT-IR spectroscopy, and Raman spectrometry [7,423,424]. Other methodologies, however, based on different analytical approaches, are being developed to broaden the detection of various arsenic species [424,425,426,427,428,429,430,431,432,433,434], often combined with challenging separation techniques [432,433,434,435]. Thus, the mechanisms underlying *i*As^(III)^-induced clinical effects serve as potential gateways for developing therapies for *i*As^(III)^ toxicity. A proteomic approach helps evaluate the mechanisms and structural changes in proteins associated with low-dose *i*As exposure, complemented by effective national and international regulations that establish a suitable threshold value for groundwater arsenic at ≤10 μg/L [436].

### 3.2. Possible Treatment

*i*As shows a high affinity for diverse types of ligands, using symmetrical *o*As^(III)^ (TMA^(III)^ and triphenyl arsine) as common ligands in coordination chemistry, although non-symmetrical *o*As^(III)^ compounds, such as 1,2-bis(dimethylarsino)benzene (diars), also act as valuable chelating ligands. However, the problem presented in this work is the inverse, potentially counteracting the toxicological effects of *i*As in living beings, where diverse metabolic paths are possible. Thus, an innovative approach employing magnetic beads modified with *Nα*-Bis(carboxymethyl)-L-lysine, a polyhistidine tag, and MB-NTA(Ni)-His6-SA enables the selective capture of *i*As binding proteins in HepG2 cells labelled by Bio-PAO(III) probes, facilitating gentle digestion by trypsin. The method is able to successfully identify 41 *i*As^(III)^-binding proteins, including those involved in cytoskeletal structure, heat shock response, transcriptional regulation, DNA damage repair, redox state regulation, mitochondrial dehydrogenase function, and protein synthesis and structure, contributing to a more comprehensive understanding of the toxic mechanisms of arsenic, and providing potential valuable insights for the prevention or treatment of arsenic-related diseases [437]. Flavonoids, some trace elements, and herbal drugs are promising natural detoxifying agents against *i*As. Other substances, such as silver nanoparticles (Ag-NPs), inhibit the As^(III)^-induced mutations, intracellular accumulation of As^(III)^ in human-hamster hybrid AL cells, suppressing the expression of specific As^(III)^-binding protein (Gal-1), and the up-regulation of antioxidant enzymes, eventually inhibiting the generation of ROS and the downstream stress-activated protein kinases/c-Jun amino-terminal kinases (SAPK/JNK) signalling pathway [438].

As previously mentioned, *i*As generates reactive free radicals that can damage DNA, at least in vitro studies using the RWPE-1 cell line, a normal human prostate epithelial cell line [439]. Nevertheless, antioxidants overcome these effects by reducing the excess of free radicals through the restoration of cellular enzymatic and non-enzymatic antioxidant activities, thereby decreasing lipid peroxidation and protein oxidation. Thus, *Nigella sativa* oil can enhance the intestine’s ability to protect against free radical-mediated *i*As toxicity by improving the antioxidant status and energy metabolism [440]. The *Gentianella acuta* treatment ameliorates the decreased maturation potential and fertilisation deficiency of *i*As^(III)^-exposed oocytes (ovocytes, female gametocytes, or germ cells involved in reproduction), also significantly inhibiting DNA damage and apoptosis and altering the H3K27me3 expression level [441]. Fruit and vegetable antioxidants (Table 3) minimise *i*As-induced genotoxicity [442]. Plant antioxidants, including those derived from the green tea extract, which contains high molecular weight polyphenols, (flavonoids such as kaempferol 3 O-α-L (600 ethyl rhamnopyranoside) and epicatechins) (Table 4), are among the most effective, showing great diversity and few side effects, but especially those with low molecular weight [443]. Thus, hydroxytyrosol, taurine, alpha-lipoic acid, ellagic acid, and thymoquinone effectively alleviate *i*As-induced neurotoxicity through their antioxidative and anti-inflammatory properties, primarily through their anti-apoptotic function via the Nrf2 and PI3/Akt/SIRT1 signalling pathways [444]. Melatonin (N-Acetyl-5-Metoxitriptamine) is a promising neuroprotective agent because it increases tissue levels of acetylcholinesterase and decreases lactate dehydrogenase and myeloperoxidase, also overcoming *i*As-induced OS and suppressing inflammation, DNA damage, and apoptosis in the foetus’s brain [445]. Similarly, free curcumin ((1E,6E)-1,7-bis(4-hydroxy-3-methoxyphenyl)-1,6-heptadiene-3,5-dione) and curcumin-loaded Poly(lactic-co-glycolic acid) nanoparticles (CUR-NP) attenuate *i*As-mediated genotoxic effects, although the same doses of nanoformulation protect better than free curcumin [446]. Curcumin also exerted inhibitory effects on inflammation and pyroptosis triggered by *i*As^(III)^, which modulates NF-κB/NLRP3 signalling pathways in the hypothalamus of ducks [447].

However, lowering *i*As toxicity is significantly improved by sequestering arsenic species, thereby overcoming the metal–protein selectivity [448] and facilitating the removal of arsenic from the body. This trend is possible and much more common using chelating agents, where the most used today is illustrated in Table 5 [449,450,451], showing high conditional stability constants with *i*As^(III)^ (Table 6) [452,453,454,455,456].

Even though all the compounds mentioned above show potential as chelating agents, some of them, such as BAL, DMSA, DMPS, and PCA, are most usually employed in acute arsenic poisoning, while others, such as ethylenediaminetetraacetic acid (EDTA) and diethylenetriamine pentaacetic acid (DTPA), show a limited practical efficacy for treating chronic arsenic poisoning [457].

Using chelating agents constitutes one effective strategy for reducing *i*As’s toxic effects, as the inert chelator–metal complex formed is excreted from the body. Diverse thiol chelators, such as *meso*-2,3-dimercaptosuccinic acid (DMSA), dimercaptopropane succinate (DMPS), 2,3-dimercapto-1-propane sulfonic acid, 2-ethyl-5,5-bis(sulfanyl)-1,3-dioxepane-4,7-dione), sodium 2,3-dimercapto-1-propane sulfonate, 2,3-dimercaptopropanol, d-penicillamine (D-(−)-2-amino-3-mercapto-3-methyl butanoic acid), and other agents can constitute suitable chelators against chronic arsenic toxicity, also assisting in normalising the GSH level [409,456]. Combining DMSA with long-chain lipophilic and hydrophobic chelators, such as monoisoamyl DMSA or monocyclohexyl DMSA, results in greater efficacy in reducing *i*As^(III)^ tissue concentrations than DMSA alone. On the other hand, DMPS chelation increases the excretion of *i*As^(III)^ in urine, even in chronic arsenic toxicity [409]. The sulphur atoms of the thiol groups (nucleophilic) join with arsenic (electrophilic). Thus, dithiols, DMSA (succimer), and BAL bind metals through –SH groups. DMSA sequesters heavy metals in the brain because it crosses the blood–brain barrier. BAL and DMSA are helpful chelating agents to sequester arsenic away from blood proteins and treat acute As^(III)^ poisoning, although they can generate hypertension as the most critical side effect. However, BAL is considerably more toxic than DMSA [457,458,459]. DMPS and DMSA have a therapeutic index higher than BAL [452]. DMSA monoesters, such as MiADMSA, are also good antidotes for As^(III)^ poisoning [460,461]. Other chelating agents (Table 5) can also achieve this, but may cause more side effects than BAL, DMSA, and DMPS. Alternative chelating agents, such as nitriloacetic acid (NTA) and DTPA, form stable metal–ligand complexes and are not used to remove *i*As. Further, NTA is a Class II carcinogen, while DTPA shows some toxicity, potentially carcinogenic [462]. Gallic acid shows antioxidant and anticancer properties, and after exposure to *i*As, gallic acid alone is more effective than when combined with MiADMSA (monoisoamyl meso-2,3-dimercaptosuccinic acid) in reducing oxidative injury. MiADMSA monotherapy provides significant therapeutic efficacy against *i*As. However, gallic acid may be a promising candidate for therapy against arsenic due to its antioxidant and anticancer properties [463]. Otherwise, the high efficiency and safety of nano-drug delivery systems are also promising alternatives to conventional chelators. The effectiveness of any chelator depends strongly on the patient’s general health situation, mode and dose of administration, and potential biotransformation. Nutritional factors also play a critical role in combating free radical species, and a balanced daily diet supplemented with essential nutrients (such as vitamins, micronutrients, and proteins) can help mitigate the severity of arsenicosis.

Flavonols, such as quercetin and kaempferol, provide tolerance to wheat plants under *i*As stress by activating antioxidant enzymes {SOD, peroxidase (POD), ascorbate peroxidase (APX)}, protecting the photosynthetic reactions from OD. Quercetin triggers the AsA renewal, and kaempferol maintains the GSH pool [464]. Further, chemicals involved in one-carbon metabolism are essential for facilitating the methylation of *i*As^(III)^ through SAM. Among them, methionine, betaine, choline, and vitamins B2 (riboflavin), B6 (pyridoxal phosphate), B9 (folate), and B12 (cobalamin) are the most important for alleviating arsenic-related adverse health outcomes by enhancing its metabolism [465]. However, a deficiency of these chemicals may impair the metabolism of As^(III)^, thereby worsening its toxicity [465,466,467,468].

## 4. Conclusions and Looking Forward

*i*As^(III)^ species and their metabolites can exhibit various chronic and genotoxic effects mediated by reactive oxidants or free radical species, including modulation of signalling pathways. There are differences in the activities of *i*As and *o*As species regarding target organ carcinogenicity and toxic and genotoxic mechanisms. The trivalent and pentavalent methylated metabolites may play some role in bladder cancer, where lower concentrations of DMA^(III,V)^ are present for promotion, leaving the question of whether arsenic needs a carcinogenic partner. Many questions remain unanswered, and future researchers must address the following significant goals.

Clarifying the methylation patterns, the relative toxicity of the methylated arsenic forms (MMA and DMA), their storage forms, and resistance mechanisms would provide insights into the relationships between entry, metabolism, interactions, bioaccumulation, and excretion of all arsenic species.

The development of unified methodologies for sample preparation and analytical procedures to monitor low levels of arsenic species and arsenic biomarkers in complex biological systems, such as hair and nails, would be beneficial compared to urine and other biological matrices.

The antagonistic and synergistic interactions of arsenic species with essential and toxic metals, diverse-sized biological molecules, and the pathways of ROS and RNS generation require straightforward elucidation.

*i*As-induced aberrant gene expression, involvement in signal transduction, carcinogenic potential, and the development of pharmacokinetic pathways represent essential areas of future knowledge that require deeper evaluation.

Evaluation of population variability, gender, and age differences in susceptibility, immunotoxicity, and immunosuppression, as well as genetic polymorphisms, requires additional confirmation. Ultimately, a worthwhile goal is to mitigate the adverse effects of arsenic exposure by developing and incorporating new, safe chelating agents.

## Figures and Tables

**Table 1:** Chemical structure of some representative compounds of arsenic.

Arsenite (*i*As^(III)^) 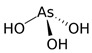	Arsenate (*i*As^(V)^) 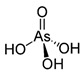
Monomethylarsonous acid (MMA^(III)^) 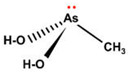	Monomethylarsonic acid (MMA^(V)^) 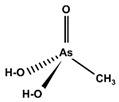
Dimethylarsinous acid (DMA^(III)^) 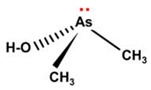	Dimethylarsinic acid (DMA^(V)^) 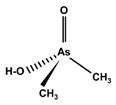
Dimethylarsine 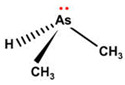	Trimethylarsine sulphide 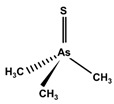
Trimethylarsine 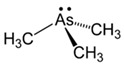	Dimethylthioarsenic acid (DMMTA^(V)^) 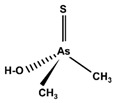
Methylarsine 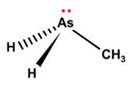	Monomethylmonothioarsinic acid (MMMTA^(V)^) 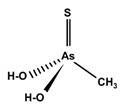
Dimethylthioarsinous acid (DMMTA^(III)^) Dimethylmonothioarsinic acid 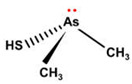	Dimethyldithioarsinic acid (DMDTA^(V)^) 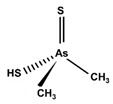
Dimethylarsine chloride 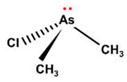	Trimethylarsonium acetate (AsBe) (Arsenobetaine) 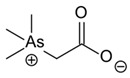
Methylarsine dichloride 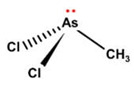	2-Hydroxyethyl (trimethyl) arsonim (Arsenocholine) (AsCho) 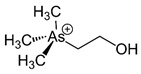
2,4,6-Triosa-1,3,5,7-tetraarsaadamantane (Arsenicin A) 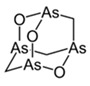	Paris green (Copper acetoarsenite) 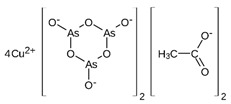

**Table 2:** Chemical structure of some representative cyclic organoarsenicals.

Salvarsan 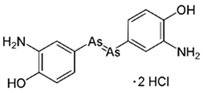	*p*-arsanilic acid 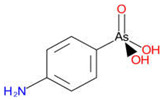
Neosalvarsan 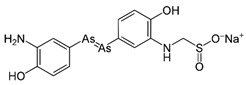	*o*-arsanilic acid 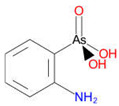
Melarsoprol 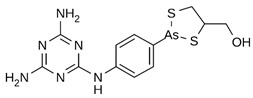	Roxarsone 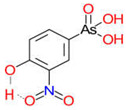
Melarsen oxide 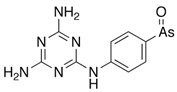	4-ureidophenyl arsenic acid 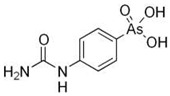
Triphenylarsine 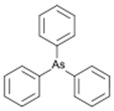	Nitarsone 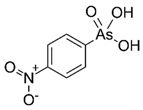
Phenyldichloroarsine 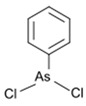	Penaphenyl arsorane 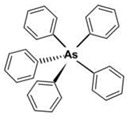
Arsabenzene 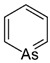	Arsole 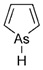
10,10′-oxybis-10*H*-phenoxarsine 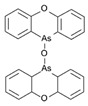	Arsenic-containing ribose derivatives 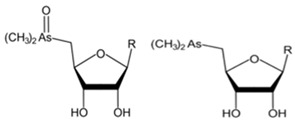

**Table 3:** Chemical structure of some representative fruit and vegetable antioxidants.

Melatonin 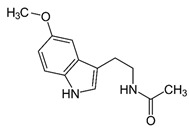	Vitamin C (ascorbic acid) 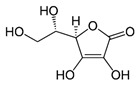
Lonidamine 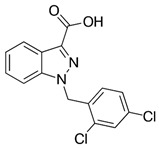	Arjunolic acid 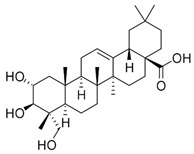
Polydatin 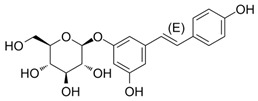	Tannins (galic acid) 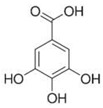
Vitamin A (retinoids, such as retinol and derivatives (retinol, retinoic acid, and carotenoids provitamin A (carotene)) 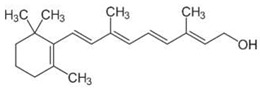	Quercetin 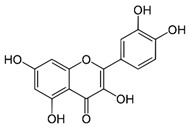
Curcumin (ceto form) (Tetrahydrocurcumin) 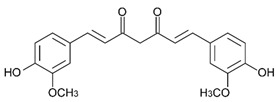	Kaempferol 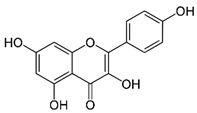
Curcumin (enol form) 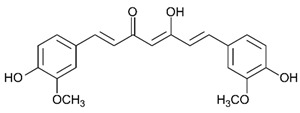	Kaempferol 3 O-a-L (600 methyl rhamnopyranoside) 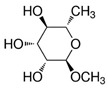
Diallyl trisulphide (DATS): (di(prop-2-en-1-yl)trisulfane) 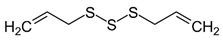	Resveratrol 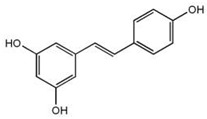
Lutein 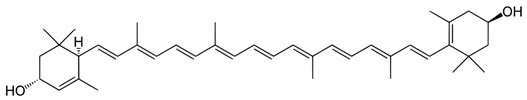	
Zeaxanthin 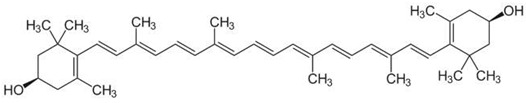	
β-carotene 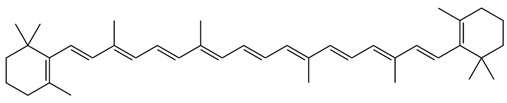	
Cryptoxantins 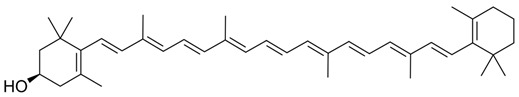	

**Table 4:** Other antioxidants.

(−)-epicatechin (EC)) (2*R*,3*R*) **((2*R*,3*S*)-2-(3,4-Dihydroxyphenyl)-3,4-dihydro-2*H*-chromene-3,5,7-triol)** 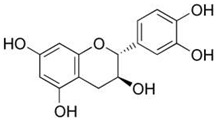	(−)-epigallocatechin (EGC) **((2*R*,3*R*)-3′,4′,5,5′,7-Pentahydroxy flavan-3-yl 3,4,5-trihydroxybenzoate)** 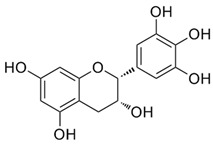
(−)-epicatechin-3-gallate (ECG) **((2*R*,3*R*)-3′,4′,5,7-Tetrahydroxyflavan-3-yl 3,4,5-trihydroxybenzoate)** 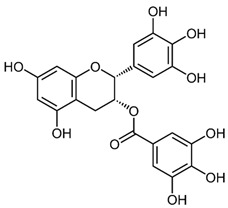	(−)-epigallocatechin-3-gallate (EGCG) **((2*R*,3*R*)-3′,4′,5,5′,7-Pentahydroxy flavan-3-yl 3,4,5-trihydroxybenzoate)** 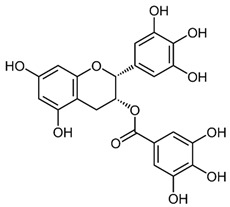
Saponins (a subclass of terpenoids) are phenolic antioxidant compounds 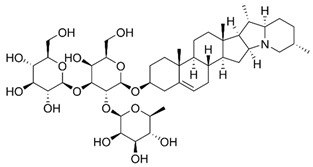	**Tannins (others)** 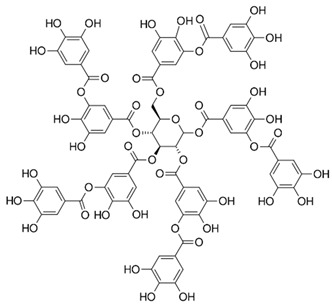

**Table 5:** Chemical structure of some representative chelating agents.

BAL 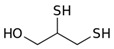	Taurine 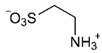
DMSA 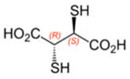	ALA 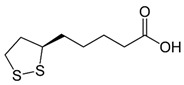
MiADMSA 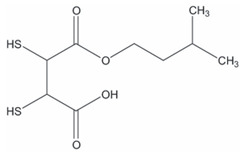	NAC 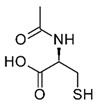
TA 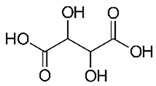	DMPS 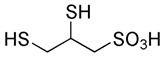
NTAP 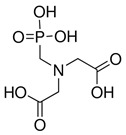	NTA2P 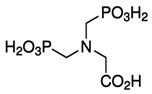
NTA3P 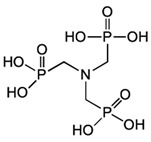	EDDS 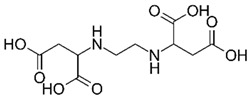

ALA: Alpha-lipoic acid (6,8-Dithiooctanoic acid); BAL: Dimercaprol (2,3-Bis(sulfanyl)propan-1-ol)); DMPS: 2,3-Dimercapto-1-propanesulfonic acid; DMSA: Dimercaptosuccinic acid ((2R,3S)-2,3-Bis(sulfanyl)butanedioic acid); EDDS: Ethylenediamine-N,N′-disuccinic acid; MiADMSA: Monoisoamyl 2,3-dimercaptosuccinic acid; NAC: N-acetyl-cysteine; NTAP: N-phosphonomethyl iminodiacetic acid; NTA2P: N,N-Bis-phosphonomethyl glycine; NTA3P: Nitrilotrimethylphosphonic acid; TA: Tartaric acid (2,3-Dihydroxybutanedioic acid); Taurine: 2-Aminoethane-1-sulfonic acid.

**Table 6:** Representative conditional stability constants between As^(III)^ and some organic ligands.

Ligand	Complex	K’ (Log β)	References
BAL	AsBAL	6.95	[452]
	AsBAL_2_	11.56	
	As_2_BAL_3_	22.73	
DMSA	AsL	9.8	[450]
	AsLH_2_	25.87	[454]
DMPS	AsL	10.68	[454]
TA	[As(OH)_2_T]^−^	6.62	[456]
DCTA	[As(OH)_2_HL]^2−^	20.67	[456]
NTAP	(L^4−^)	20.91	[455]
NTA2P	(L^5−^)	24.78	[455]
NTA3P	(L^6−^)	32.52	[455]

The abbreviations mean the same as in Table 5.

## Data Availability

No new data were created or analyzed in this study. Data sharing is not applicable to this article.

## Appendix A

**Table A1:** Names, abbreviations, and formulas of other arsenic compounds and related biomolecules.

Chemical Species	Abbreviation	Chemical Formula
Monomethyldithioarsonic acid	MMDTA^(V)^	CH_3_AsS(SH)(OH)
Arsine	−	AsH_3_
Trimethylarsine oxide	TMAO^(V)^	(CH_3_)_3_AsO
Arsenic triglutathione	ATG^(III)^	As(SG)_3_
Monomethylarsino-diglutathione	MMADG^(III)^	CH_3_As(SG)_2_
Dimethylarsino-glutathione	DMAG^(III)^	(CH_3_)_2_As(SG)
Dimethylarsenic radical	−	(CH_3_)_2_As^•^
Dimethylarsenic peroxyl radical	−	(CH_3_)_2_AsOO^•^
Reduced glutathione	GSH	
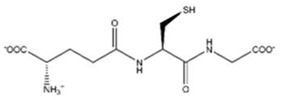
Oxidised glutathione	GSSG	
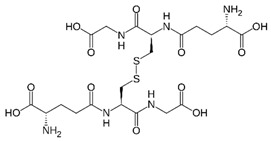
S-adenosylmethionine	SAM	
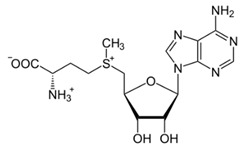
S-adenosyl-L-homocysteine	SAH	
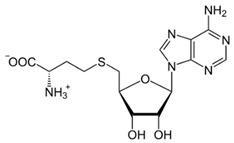
Lipoic acid		
